# Knockdown of Yin Yang 1 enhances anticancer effects of cisplatin through protein phosphatase 2A-mediated T308 dephosphorylation of AKT

**DOI:** 10.1038/s41419-018-0774-8

**Published:** 2018-07-03

**Authors:** Lu Zhao, Ran Li, Ye-Hua Gan

**Affiliations:** 1 Central Laboratory, Peking University School and Hospital of Stomatology, 22 Zhongguancun Avenue South, Haidian District, Beijing, 100081 P. R. China; 2 Department of Oral & Maxillofacial Surgery, Peking University School and Hospital of Stomatology, 22 Zhongguancun Avenue South, Haidian District, Beijing, 100081 P. R. China; 3 National Engineering Laboratory for Digital and Material Technology of Stomatology, Beijing Key Laboratory of Digital Stomatology, Peking University School and Hospital of Stomatology, 22 Zhongguancun Avenue South, Haidian District, Beijing, 100081 P. R. China; 4 Department of Oral & Maxillofacial Surgery, Qingdao Stomatological Hospital, Qingdao, P. R. China

## Abstract

Cisplatin is still one of the first-line drugs for chemotherapy of head and neck squamous cell carcinoma (HNSCC) and shows a survival advantage for HNSCC. However, a substantial proportion of HNSCC eventually becomes resistance to cisplatin and the underlying mechanisms remain to be fully understood. Yin Yang 1 (YY1) is a multifunctional protein regulating both gene transcription and protein modifications and also plays a role in chemotherapy resistance. Here, we reported that knockdown of YY1 by lentivirus-mediated short hairpin RNA or tetracycline-inducible short hairpin RNA enhanced cisplatin-induced apoptosis and inhibition of cell proliferation, migration and invasion in the HNSCC cell lines, and inhibition of the xenograft tumor growth. The underlying mechanisms were revealed that knockdown of YY1 downregulated both S473 and T308 phosphorylation of AKT (protein kinase B), which was mainly responsible for cisplatin resistance, whereas overexpression of YY1 upregulated both S473 and T308 phosphorylation. Cisplatin upregulated YY1 mRNA and protein expression and both S473 and T308 phosphorylation of AKT. In the presence of cisplatin, knockdown of YY1 not only blocked cisplatin-induced increase in S473 and T308 phosphorylation of AKT, but still downregulated T308 phosphorylation. Moreover, protein phosphatase 2A (PP2A) antagonist, okadaic acid, upregulated T308, but not S473, phosphorylation, and simultaneously abolished YY1 knockdown-mediated enhancement of cisplatin-induced inhibition of cell proliferation. In addition, knockdown of YY1 promoted PP2A activity through upregulating mRNA and protein expressions of PP2A catalytic subunit alpha (PPP2CA) through the binding of YY1 in the promoter of PPP2CA. Conversely, activating PP2A by forskolin also promoted YY1 degradation and subsequently inhibited T308 phosphorylation. These results suggested that knockdown of YY1 enhanced anticancer effects of cisplatin through PP2A mediating T308 dephosphorylation of AKT, and that targeting YY1 or PP2A would enhance the efficiency of cisplatin chemotherapy in treatment of HNSCC.

## Introduction

Head and neck squamous cell carcinoma (HNSCC) comprises 90% of head and neck cancers and has high recurrence rate associated with resistance to chemotherapy and lowest 5-year survival rate in any major cancers^1,2^. Cisplatin, a DNA-damaging agent, exerts anticancer effects by causing inhibition of DNA synthesis^3^ and is still one of the first-line drugs for chemotherapy of HNSCC^4,5^. Few improvements in chemotherapeutic treatment of HNSCC have been made in last 30 years, and molecular basis of acquired chemoresistance of cisplatin in HNSCC remains largely unknown^6^ and the chemoresistance is a major complication for cisplatin chemotherapy^7,8^.

AKT (protein kinase B), which is a serine/threonine-specific protein kinase, plays an important role in cisplatin chemoresistance^9–11^. Phosphorylated AKT is the activated form of AKT and AKT activity is regulated by reversible phosphorylation^12^. AKT is phosphorylated at two sites, serine 473 (S473) and threonine 308 (T308), which are mainly catalyzed by mammalian target of rapamycin complex 2 (mTORC2)^13^ and 3-phosphoinositide dependent protein kinase 1 (PDK1)^14^, respectively. Dephosphorylation or inactivation of AKT is mainly mediated by pleckstrin homology domain leucine-rich repeat protein phosphatase (PHLPP)^15,16^, which removes phosphate group from S473, and protein phosphatase 2A (PP2A), which removes phosphate group from T308^17^. Phosphoinositide 3-kinase (PI3K) phosphorylates phosphatidylinositol-4,5-biphosphate (PIP2) into phosphatidylinositol-3,4,5-triphosphate (PIP3) at cell membrane, which binds to pleckstrin homology domain of AKT and PDK1 leading to T308 phosphorylation and also activates mTORC2 to phosphorylate S473^18,19^. T308 phosphorylation is necessary and also sufficient for AKT activation^20–22^. Further understanding of mechanisms underlying AKT-mediating cisplatin chemoresistance was still clinically and theoretically important and would help improve chemotherapeutic treatment of HNSCC patients.

AKT activation can also be regulated by Yin Yang 1 (YY1)^23^. YY1, a transcription factor, can either activate^24,25^ or repress^26,27^ the expression of genes. Recent studies revealed a proliferative role of YY1 in carcinogenesis^28–34^. High level of YY1 correlates with poor prognoses of many types of cancers^35^. However, the expression and function of YY1 in HNSCC remain to be explored. YY1 also directly interacts with several important cancer-related regulators including AKT^23^. Moreover, overexpression of YY1 confers cancer cells with resistance in chemotherapies^36–39^. Therefore, we hypothesized that YY1 might be involved in AKT-mediated cisplatin resistance.

Protein phosphatase 2A (PP2A), as an important negative regulator of AKT phosphorylation, is also involved in cisplatin resistance. PP2A is a ubiquitously expressed, highly conserved heterotrimeric serine/threonine phosphatase, and regulates cell functions by dephosphorylating many critical cellular molecules including AKT^40–43^. PP2A is composed of a scaffold subunit and a catalytic subunit to form dimeric core enzyme, and a regulatory subunit binding to the dimeric core enzyme to form functional holoenzyme^44^. The catalytic subunit activity represents PP2A activity^45^ and it has two isoforms, alpha and beta, and the alpha isoform is encoded by the protein phosphatase 2 catalytic subunit alpha (*PPP2CA*)^46^. PP2A activity is mainly regulated by PPP2CA^47–49^. However, the regulation of PPP2CA expression remains to be fully understood^50–52^. Upregulation of PP2A activity can sensitize cancer cells to cisplatin^53–55^. Therefore, we further hypothesized that PP2A might also be involved in AKT-mediated cisplatin resistance.

In this study, we showed that knockdown of YY1 enhanced cisplatin-mediated anticancer effects through AKT dephosphorylation at T308 by upregulation of PP2A activity.

## Results

### YY1 highly expressed in the tongue cancer and knockdown of YY1 enhanced cisplatin-induced inhibition of cell proliferation, migration and invasion, and cisplatin-induced inhibition of xenograft tumor growth

**Fig. 1:**
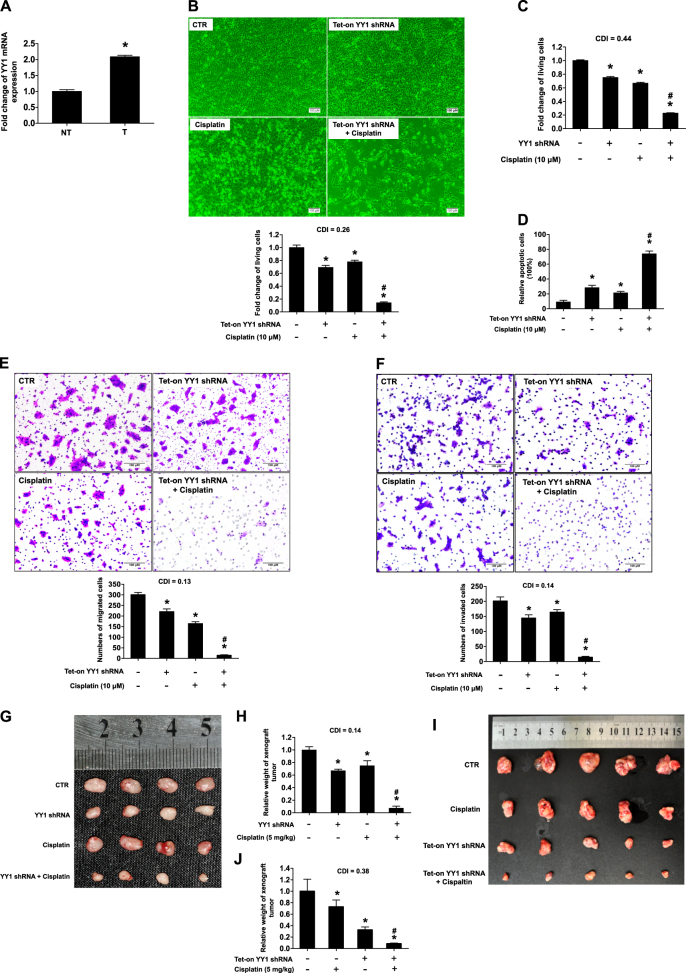
YY1 highly expressed in the tongue cancer and knockdown of YY1 enhanced cisplatin-induced inhibition of cell proliferation, migration and invasion, and cisplatin-induced inhibition of xenograft tumor growth . **a** YY1 mRNA expression in tongue cancer specimens. The mRNA expressions of YY1 in the tumor tissue and adjacent normal tissue of 37 cases were assessed by real-time PCR, *P < 0.05 (*t*-test, *n* = 37), NT, adjacent normal tissue; T, tumor. **b**, **c** Knockdown of YY1 enhanced cisplatin-induced cell proliferation inhibition. **b** CAL27 cells stably transfected with Tet-on YY1 shRNA were exposed to either doxycycline (100 nM) or cisplatin (10 μM) or both for 48 h and subjected to CCK-8 assay, *n* = 4; Microphotographs of CAL27 cells after different treatment, scale bar: 100 μm. **c** CAL27 cells stably transfected with YY1 shRNA or scramble shRNA were exposed to 10 μM cisplatin or not for 48 h and subjected to CCK-8 assay, *n* = 4. **d** Knockdown of YY1 enhanced cisplatin-induced cell apoptosis. CAL27 cells stably transfected with Tet-on YY1 shRNA were exposed to either doxycycline (100 nM) or cisplatin (10 μM) or both for 48 h and subjected to DAPI, scale bar: 100 μm. Cell counting of apoptotic cells from four separated fields and the rate of apoptotic cells was quantified, *n* = 4. **e**, **f** Knockdown of YY1 enhanced cisplatin-induced inhibition of cell migration and invasion. CAL27 Cells stably transfected with Tet-on YY1 shRNA were exposed to either doxycycline (100 nM) or cisplatin (10 μM) or both for 48 h. Transwell migration (**e**) or invasion (**f**) of the cells were photographed under a light microscope, scale bar: 100 μm. Migrated cells were counted from four separated fields, *n* = 4. **g**–**j** Knockdown of YY1 enhanced cisplatin-induced inhibition of xenograft tumor growth. Photograph and weights of xenograft tumors. **g**, **h** We injected nude mice with twice the amount of CAL27 cells stably transfected with YY1 shRNA lentivirus compared with CAL27 cells stably transfected with scramble shRNA lentivirus at groin and to make sure that the initial size of xenografts was equal before intraperitoneal injection of cisplatin, *n* = 4. **i**, **j** We injected nude mice with equal CAL27 cells stably transfected with Tet-on YY1 shRNA at axilla. We supplied with normal water or Dox (1 mg/ml) containing water and injected cisplatin or not, *n* = 5. All data (mean ± SD of four separated experiments) were presented as folds of the control group. **b**–**j** One-way ANOVA: *P < 0.05 vs. control group, ^#^*P < *0.05 vs. YY1 knockdown or cisplatin group

YY1 mRNA expression was upregulated in tongue cancer compared with that in the adjacent normal tissue (Fig. 1a). To evaluate whether YY1 was required for cisplatin resistance in HNSCC cells, we used Tet-on inducible shRNA or lentiviral shRNA to knockdown YY1 in CAL27 cells. Knockdown of YY1 by both knockdown systems significantly enhanced cisplatin-induced apoptosis and inhibition of cell proliferation, compared with YY1 knockdown or cisplatin group, which showed only slightly inhibition of cell proliferation (Fig. 1b–d and Supplemental Figs. 2–3). YY1 knockdown was confirmed by Western blot (Supplemental Fig. 1).

Knockdown of YY1 appeared to synergistically enhance cisplatin-induced inhibition of cell proliferation, since the CDI were 0.26 and 0.44 (much less than 0.7) for the two knockdown systems, respectively. Similar effects were also observed when YY1 was knocked down by siRNA in SCC9 and WSU-HN6 cells (Supplemental Figs. 4–5). In addition, knockdown of AKT by siRNA or inhibition of AKT activation by perifosine or LY294002 also synergistically enhanced cisplatin-induced inhibition of cell proliferation, similar to that of knockdown of YY1 did on cisplatin-induced inhibition of cell proliferation (Supplemental Figs. 6–8). In contrast, cells with constitutive activation of AKT showed resistant to cisplatin (Supplemental Figure 12).

Knockdown of YY1 also significantly promoted cisplatin-induced inhibition of cell migration and invasion (Fig. 1e, f). Moreover, we confirmed in nude mice that knockdown of YY1 synergistically enhanced cisplatin-induced inhibition of xenograft tumor growth. The weights of xenografts in the group received combinational treatment with cisplatin and YY1 knockdown by tetracycline-induced shRNA or lentivirus-mediated shRNA was significantly lower than that of groups received non-treatment, or treatment with only knockdown of YY1 or cisplatin (Fig. 1g–j and Supplemental Figures 9 and 10).

### Cisplatin upregulated AKT phosphorylation through YY1

**Fig. 2:**
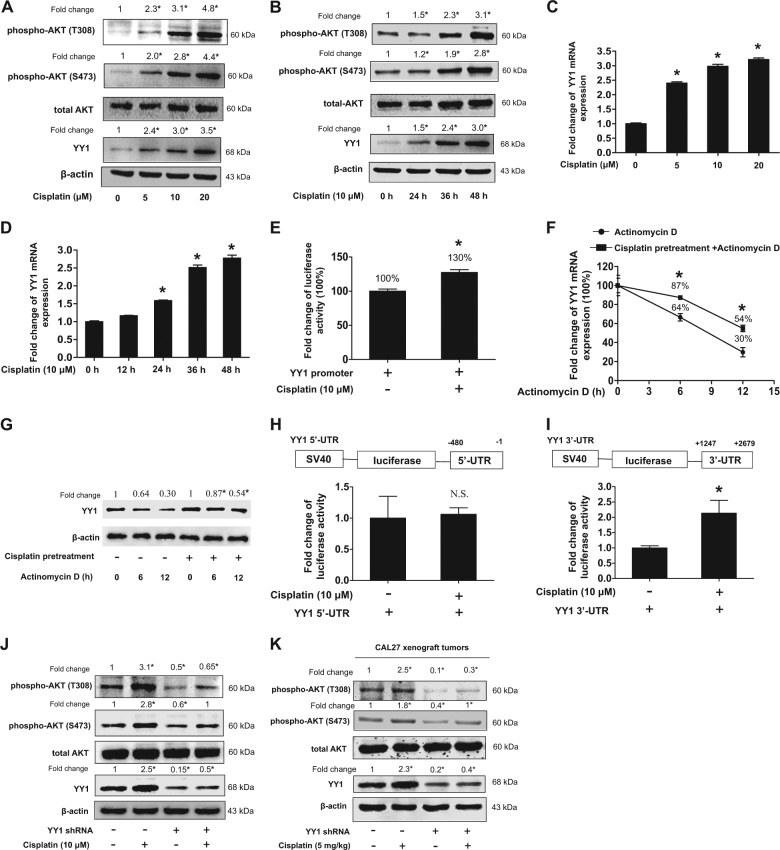
Cisplatin upregulated AKT phosphorylation through YY1. **a**, **b** Expressions of YY1 and AKT phosphorylation (S473 and T308) after treatment with cisplatin. **a** CAL27 cells were exposed to different dosage of cisplatin for 48 h. Total protein was extracted and subjected to Western blot for analysis, one-way ANOVA: *P < 0.05 vs. the control group, *n* = 3. **b** CAL27 cells were exposed to cisplatin (10 μM) and protein was collected at indicated time points and then subjected to Western blot analysis, one-way ANOVA: *P < 0.05 vs. the control group, *n* = 3. **c**, **d** Expression of YY1 mRNA after treatment with cisplatin. **c** CAL27 cells were exposed to different dosage of cisplatin for 30 h. mRNA expressions were quantitated by real-time PCR, one-way ANOVA: *P < 0.05 vs. the control group, *n* = 3. **d** CAL27 cells were exposed to cisplatin (10 μM) and mRNA was collected at indicated time points. mRNA expressions were quantitated by real-time PCR, one-way ANOVA: *P < 0.05 vs. the control group, *n* = 3. **e** Cisplatin upregulated YY1 promoter activity. CAL27 cell was transfected with YY1 promoter (−1500/+40) and then treated with cisplatin for 24 h. Total protein were extracted and subjected to luciferase assay and normalized by total protein concentration. *t*-test, *P < 0.05 (*n* = 6). **f**, **g** Effect of cisplatin on YY1 mRNA stability. CAL27 cells were pretreated with 10 μM cisplatin or not for 24 h. At *t* = 0, 1 μM actinomycin D was added to arrest further transcription, YY1 mRNA (**f**) and protein (**g**) expressions were quantitated by real-time PCR and Western blot after 6 h and 12 h. *t*-test, *P < 0.05 vs. actinomycin D at 6 h and 12 h separately (*n* = 3). **h**, **i** Effect of cisplatin on YY1 3′-UTR and 5′-UTR luciferase activity. CAL27 cells transfected with YY1 5′-UTR luciferase vector (**h**) and 3′-UTR luciferase vector (**i**) for 24 h and exposed to 10 μM cisplatin or not for another 24 h and total protein were extracted and subjected to luciferase assay and normalized by total protein concentration, *t*-test, *P < 0.05, N.S.: no statistical differences (*n* = 6). **j**, **k** Knockdown of YY1 blocked cisplatin-induced T308 and S473 phosphorylation of AKT. **j** CAL27 cells stably transfected with YY1 shRNA or scramble shRNA lentivirus and exposed to cisplatin or not for 48 h. Total protein was extracted and subjected to Western blot analysis. (**k**) Proteins were extracted from nude mice xenograft samples and subjected to Western blot analysis. **a**, **b**, **g**, **j**, **k** The target bands were exposed, and densitometry was performed as fold change ratio from the mean of three independent experiments, one-way ANOVA: *P < 0.05 vs. the control group. All data (mean ± SD of three or six separated experiments) were presented as folds of the control group

As shown in Fig. 2a, b, cisplatin upregulated both YY1 protein expression and AKT phosphorylation at T308 and S473 in a dose- and time-dependent manner. Cisplatin also upregulated YY1 mRNA expression in a dose- and time-dependent manner (Fig. 2c, d). However, cisplatin only upregulated YY1 promoter activity by about 1.3 folds at 24 h (Fig. 2e), but upregulated YY1 mRNA expression by about 1.46 folds at 24 h (Fig. 2d), implying that cisplatin might also stabilize YY1 mRNA to upregulate YY1 mRNA. It was confirmed in Fig. 2f, g, which showed that cisplatin pretreatment for 24 h increased YY1 mRNA and protein by 23% compare with that of the group without cisplatin pretreatment in the presence of actinomycin D for 6 h or 12 h. Moreover, cisplatin upregulated the luciferase activity of the reporter containing 3′-untranslated region (UTR), but not 5′-UTR, of YY1 mRNA (Fig. 2h, i), suggested that 3′-UTR was involved in cisplatin-induced YY1 mRNA stabilization.

Moreover, knockdown of YY1 by lentivirus-mediated shRNA totally blocked cisplatin-induced T308 and S473 phosphorylation of AKT in CAL27 cells and also in CAL27 xenograft tumors (Fig. 2j, k). We also confirmed that knockdown of YY1 by tetracycline-inducible shRNA or lentivirus-mediated shRNA downregulated T308 and S473 phosphorylation in CAL27 cells (Supplemental Figure 13), and that knockdown of YY1 by siRNA also similarly downregulated T308 and S473 phosphorylation in SCC9 and WSU-NH6 cells (Supplemental Figure 14). Conversely, overexpression of YY1 upregulated T308 and S473 phosphorylation in CAL27, SCC9, and WSU-NH6 cells (Supplemental Figures 15–16).

### PP2A-mediated T308 dephosphorylation was responsible for YY1 knockdown-induced enhancement of anti-proliferative effect of cisplatin

**Fig. 3:**
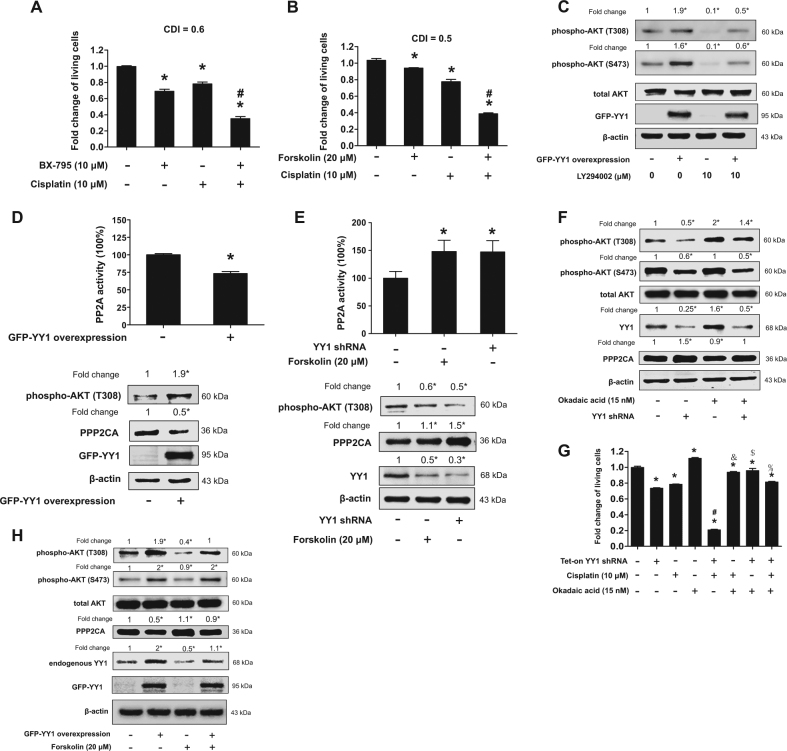
PP2A-mediated T308 dephosphorylation was responsible for YY1 knockdown-induced enhancement of anti-proliferative effect of cisplatin. **a** BX-795 enhanced cisplatin-induced cell proliferation inhibition. CAL27 cells were treated with BX-795 (10 μM) or cisplatin (10 μM) or both for 48 h and subjected to CCK-8 assay, (*n* = 4). One-way ANOVA: *P < 0.05 vs. control group; ^*#*^*P* < 0.05 vs. BX-795 or cisplatin group. **b** Forskolin enhanced cisplatin-induced cell proliferation inhibition. CAL27 cells were treated with forskolin (20 μM) or cisplatin (10 μM) or both for 48 h and subjected to CCK-8 assay, (*n* = 4). One-way ANOVA: *P < 0.05 vs. control group; ^*#*^*P* < 0.05 vs. forskolin or cisplatin group. **c** YY1 overexpression partially rescued LY294002-induced AKT phosphorylation inhibition. CAL27 cells infected with lentivirus carrying GFP empty vector or PLVX-GFP-YY1 vector and exposed to LY294002 for 48 h. Total protein was extracted and subjected to Western blot analysis. **d** YY1 overexpression inhibited PP2A activity and PPP2CA protein expression but upregulated AKT T308 phosphorylaiton. CAL27 cells infected with lentivirus carrying GFP empty vector or PLVX-GFP-YY1 vector. Whole-cell lysates were immunoprecipitated with PPP2CA antibody and subjected to PP2A immunoprecipitation phosphatase assay. Protein was extracted from the same samples and subjected to Western blot. *t-*test, *P < 0.05 (*n* = 4). **e** Knockdown of YY1 upregulated PP2A activity and PPP2CA protein expression but inhibited AKT T308 phosphorylation. CAL27 cells stably transfected with scramble shRNA or YY1 shRNA lentivirus. Cells treated with forskolin as a positive control. The experiment was performed similar to that in (**d**), one-way ANOVA: *P < 0.05 (*n* = 4). **f** Okadaic acid rescued YY1 knockdown-induced AKT T308 phosphorylation inhibition but failed to rescued YY1 knockdown-induced AKT S473 phosphorylation inhibition. CAL27 cells stably transfected with scramble shRNA or YY1 shRNA and treated with okadaic acid (15 nM) or not for 48 h. Total proteins were extracted and subjected to Western blot analysis. **g** Okadaic acid abolished YY1 knockdown-induced or cisplatin-induced or their combination-induced inhibition of cell proliferation. CAL27 cells stably transfected with Tet-on YY1 shRNA were exposed to either doxycycline (100 nM) or okadaic acid (15 nM) or cisplatin (10 μM) or both for 48 h and subjected to CCK-8 assay (*n* = 4), one-way ANOVA: *P < 0.05 vs. control group; ^#^*P* < 0.05 vs. YY1 knockdown or cisplatin group; ^*&*^*P* < 0.05 vs. cisplatin group; ^*$*^*P* < 0.05 vs. YY1 knockdown group; ^*%*^*P < *0.05 vs. the combination of cisplatin and YY1 knockdown group. **h** Forskolin blocked YY1 overexpression-induced upregulation of AKT T308 phosphorylation but failed to blocked YY1 overexpression-induced upregulation of AKT S473 phosphorylation. CAL27 cells infected with GFP empty or PLVX-GFP-YY1 lentivirus and treated with forskolin (20 μM) or not for 48 h. Total proteins were extracted and subjected to Western blot analysis. **c**, **d**, **e**, **f**, **h** The target bands were exposed, and densitometry was performed as fold change ratio from the mean of three independent experiments, one-way ANOVA (**c**, **e**, **f**, **h**) or *t-*test (**d**), *P < 0.05 vs. control group. (Bars: mean ± SD)

T308 phosphorylation was required for AKT-mediated cisplatin resistance, since BX-795, a specific inhibitor for PDK1, which is responsible for AKT phosphorylation at T308, or forskolin, an agonist for PP2A, which is mainly responsible for AKT dephosphorylation at T308, both synergistically enhanced cisplatin-induced inhibition of cell proliferation, similar to knockdown of YY1 (Fig. 3a, b and Supplemental Figures 17 and 18). Expectedly, BX-795 only inhibited T308 phosphorylation, but did not affect S473 phosphorylation (Supplemental Figure 19). In addition, LY294002, an inhibitor for PI3K, which promotes AKT phosphorylation at T308 and S473^18,19^, also expectedly inhibited T308 and S473 phosphorylation, and overexpression of YY1 could partially rescue LY294002-induced inhibition of T308 and S473 phosphorylation of AKT (Fig. 3c), suggesting that overexpression of YY1 might inhibit phosphatases of AKT.

We then tested whether YY1 could affect PP2A activity. As shown in Fig. 3d, overexpression of YY1 inhibited PP2A activity by about 30%, compared to that of control group, and upregulated AKT phosphorylation (T308) by 1.9 folds, and downregulated PPP2CA protein expression by about 50%. Conversely, knockdown of YY1 upregulated PP2A activity by 150%, similarly to the effect of forskolin at 20 μM on PP2A activity (Fig. 3e), and correspondingly downregulated AKT phosphorylation (T308) by 50%, and upregulated PPP2CA protein expression by 1.5 folds, compared to that of control group (Fig. 3e). Moreover, PP2A antagonist, okadaic acid, significantly upregulated YY1 protein expression and T308 phosphorylation, but did not affect S473 phosphorylation, and totally rescued YY1 knockdown-induced downregulation of T308 phosphorylation, but not S473 phosphorylation (Fig. 3f). More importantly, okadaic acid abolished YY1 knockdown-induced or cisplatin-induced or their combination-induced inhibition of cell proliferation (Fig. 3g and Supplemental Figure 20). Combination of okadaic acid and YY1 knockdown showed antagonistic effects, as CDI was 1.15 (Supplemental Figure 21).

We also noticed that forskolin slightly upregulated PPP2CA protein expression, but significantly downregulated YY1 protein expression (Fig. 3e,h), whereas okadaic acid slightly downregulated PPP2CA protein expression, but significantly upregulated YY1 protein expression (Fig. 3f). As shown in Fig. 3h, forskolin significantly inhibited T308 phosphorylation (to 40% of the control) and only slightly inhibited S473 phosphorylation (to 90% of the control), and totally blocked YY1 overexpression-induced upregulation of T308 phosphorylation, but not S473 phosphorylation, although overexpression of YY1 induced both T308 and S473 phosphorylation of AKT. In addition, overexpression of YY1 also induced endogenous YY1 protein expression and simultaneously inhibited PPP2CA protein expression (Fig. 3h), whereas forskolin also almost blocked YY1 overexpression-induced upregulation of endogenous YY1 protein expression (Fig. 3h). All these results suggested that knockdown of YY1 enhanced anti-proliferative effect of cisplatin through PP2A/PPP2CA-mediated T308 dephosphorylation.

### YY1 regulated PPP2CA expression through binding to PPP2CA promoter

**Fig. 4:**
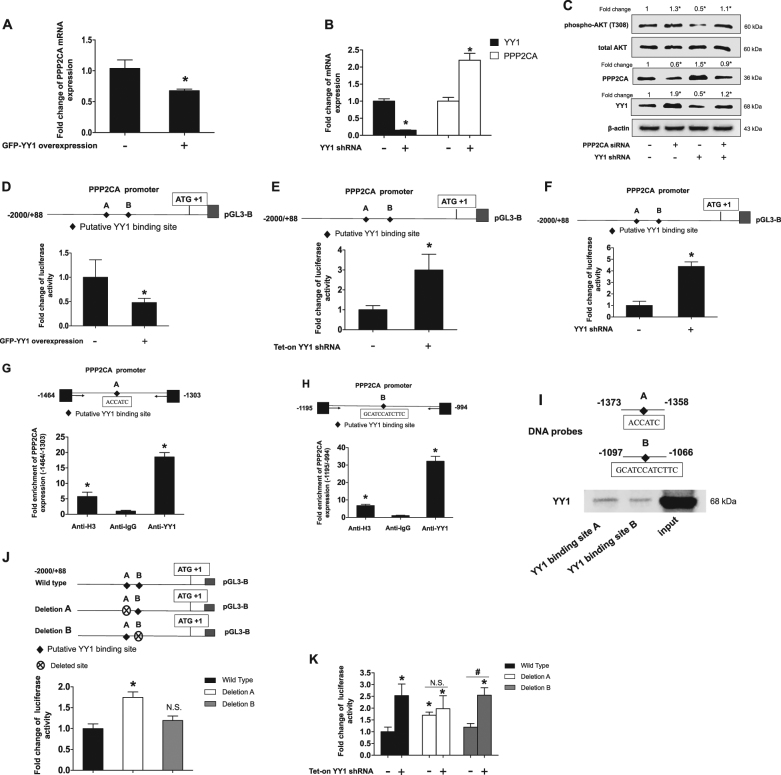
YY1 regulated PPP2CA expression through binding to PPP2CA promoter. **a** YY1overexpression inhibited PPP2CA mRNA expression. CAL27 cells infected with GFP empty vector or PLVX-GFP-YY1 lentivirus and PPP2CA mRNA expressions were quantitated by real-time PCR. *t-*test, *P < 0.05 (*n* = 3). **b** Knockdown of YY1 upregulated PPP2CA mRNA expression. CAL27 cells stably transfected with scramble shRNA or YY1 shRNA and YY1 and PPP2CA mRNA expressions were quantitated by real-time PCR. *t-*test, *P < 0.05 (*n* = 3). **c** Knockdown of PPP2CA rescued YY1 knockdown-induced AKT T308 phosphorylation inhibition. Specific siRNA for PPP2CA was transfected into CAL27 cells that stably knockdown of YY1 for 48 h. Total proteins were extracted and subjected to Western blot analysis. The target bands were exposed, and densitometry was performed as fold change ratio from the mean of three independent experiments, one-way ANOVA: *P < 0.05 vs. control group. **d** YY1 overexpression inhibited PPP2CA promoter activity. PPP2CA promoter was transfected in CAL27 cells which stably infected with either PLVX-GFP-YY1 or GFP empty vector lentivirus for 24 h. **e**, **f** Knockdown of YY1 upregulated PPP2CA promoter activity. **e** CAL27 cells stably transfected with Tet-on YY1 shRNA and pretreated with doxycycline for 24 h and then transfected with PPP2CA promoter for another 24 h. **f** PPP2CA promoter was transfected into CAL27 cells which stably infected with either YY1 shRNA or scramble shRNA lentivirus for 24 h. **d**–**f** Total proteins were extracted and subjected to luciferase assay and normalized by total protein concentration. Data (mean ± SD) were presented as folds of control group. *t*-test, *P < 0.05 (*n* = 9). **g**, **h** YY1 bound to PPP2CA promoter in vivo. ChIP assays were performed in CAL27 cells with anti-YY1, anti-H3 or anti-IgG antibodies and with primers amplifying the −1464/−1303 and −1195/−994 region of the PPP2CA promoter containing YY1-binding sites **A** or **B**, respectively. ChIP samples was assessed by real-time PCR, data (mean ± SD) were presented as folds of anti-IgG group, one-way ANOVA: *P < 0.05 vs. Anti-IgG (*n* = 3). (**I**) YY1 bound to PPP2CA promoter in vitro. DAPA was performed using PPP2CA promoter −1373/−1358 and −1097/−1066 regions with YY1 binding site **A** and **B** respectively as DNA probes. The membrane was detected with YY1 antibody. **j**, **k** Deletion of YY1-binding site A almost abolished YY1 knockdown-induced upregulation of PPP2CA promoter activity. Deleted YY1-binding site was represented by crossed circle. Reporter constructs were transfected in CAL27 cells which stably transfected with Tet-on YY1 shRNA and treated with doxycycline or not for 24 h. Data (mean ± SD) were presented as folds of the non-treatment wild type group, one-way ANOVA: *P < 0.05 vs. non-treatment of wild type group; ^*#*^*P* < 0.05 as indicated; N.S.: no statistical differences (*n* = 6)

Overexpression of YY1 downregulated PPP2CA mRNA expression, whereas knockdown of YY1 upregulated PPP2CA mRNA expression (Fig. 4a, b). Interestingly, knockdown of PPP2CA conversely upregulated YY1 protein expression and T308 phosphorylation, and totally blocked YY1 knockdown-induced upregulation of PPP2CA and downregulation of T308 phosphorylation (Fig. 4c), suggesting that PPP2CA mediated YY1 regulating T308 phosphorylation. We further confirmed that YY1 upregulated PPP2CA expression through activating transcription of PPP2CA, since overexpression of YY1 inhibited PPP2CA promoter activity, whereas knockdown of YY1 upregulated PPP2CA promoter activity (Fig. 4d–f). According to YY1-binding consensus and prediction by JASPAR database (an open-access database of curated, non-redundant transcription factor binding profiles), there were two putative binding sites with highest scores and were designated as YY1-binding site A and B located at −1368/−1363 and −1097/−1066, respectively, in *PPP2CA* promoter. As shown in Fig. 4g, h, YY1 could bind to the both putative binding site A and B. Expected DNA fragments of 161 bp and 201 bp were significantly amplified from the chromatin precipitated by anti-YY1 antibody, but not by anti-IgG antibody (Supplemental Figure 22). We also confirmed the binding of YY1 to *PPP2CA* promoter using DNA-affinity precipitation assay, since YY1 was detected in precipitates pulled down by DNA probes containing YY1 binding site A or B (Fig. 4i), Moreover, deletion of YY1-binding site A upregulated PPP2CA promoter activity by 1.7 folds, and even almost abolished YY1 knockdown-induced upregulation of PPP2CA promoter activity, whereas deletion of YY1-binding site B failed to produce the similar effects (Fig. 4j, k).

### Activation of PP2A induced degradation of YY1 protein

**Fig. 5:**
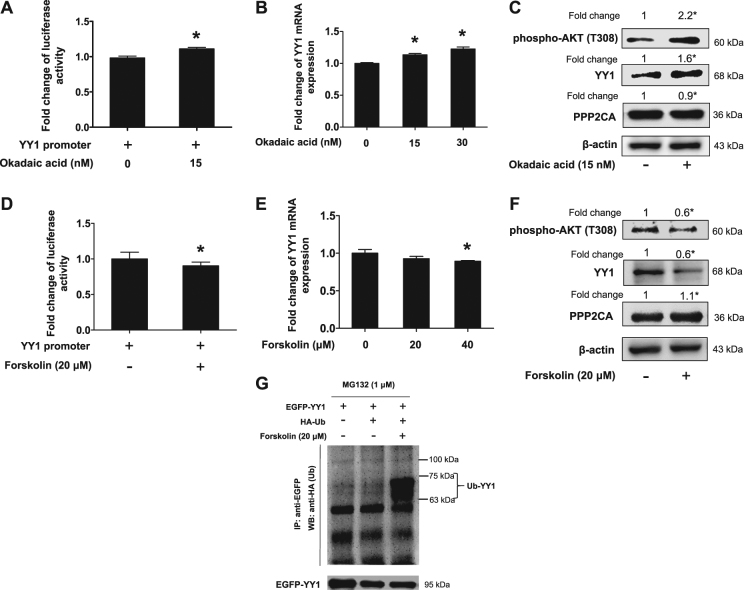
Activation of PP2A induced degradation of YY1 protein. **a** Okadaic acid slightly upregulated YY1 promoter activity. CAL27 cells transfected with YY1 promoter plasmid and treated with okadaic acid or not for 24 h. Total proteins were extracted and subjected to luciferase assay and normalized by total protein concentration. *t*-test, *P < 0.05 (*n* = 9). **b**, **c** Okadaic acid slightly upregulated YY1 mRNA but significantly upregulated YY1 protein expression. CAL27 cells were exposed to okadaic acid for 48 h, mRNA expression was quantitated by real-time PCR, one-way ANOVA: *P < 0.05 (*n* = 3) **b**; total protein was subjected to Western blot analysis **c**, *t*-test, *P < 0.05 (*n* = 3). **d** Forskolin slightly downregulated YY1 promoter activity. CAL27 cells transfected with YY1 promoter plasmid and treated with forskolin or not for 24 h. Total protein was subjected to luciferase assay and normalized by total protein concentration. *t*-test, *P < 0.05 (*n* = 9). **e**, **f** Forskolin slightly downregulated YY1 mRNA but significantly downregulated YY1 protein expression. CAL27 cells were treated with forskolin for 48 h, mRNA expression was quantitated by real-time PCR, one-way ANOVA: *P < 0.05, *n* = 3 (**e**); total protein was subjected to Western blot analysis, *t*-test, *P < 0.05, *n* = 3 (**f**). **g** Forskolin induced ubiquitination of YY1 expression. 293 T cells were transfected with HA-ubiquitin or GFP-YY1 or both and transfected cells were exposed to forskolin or not for 48 h. Whole-cell lysates were immunoprecipitated with GFP antibody and the immunocomplexes were subjected to Western blot with HA antibody. **c**, **f** The target bands were exposed, and densitometry was performed as fold change ratio from the mean of three independent experiments, *t*-test, *P < 0.05. (Bars: mean ± SD)

We then investigated whether PP2A affected YY1 protein expression through transcription of YY1. As shown in Fig. 5a, b, PP2A antagonist okadaic acid only slightly upregulated YY1 promoter activity and mRNA expression both about by 1.1 folds, but okadaic acid upregulated YY1 protein expression by 1.6 folds (Fig. 5c). Conversely, PP2A agonist forskolin slightly downregulated YY1 promoter activity and mRNA expression both by 10% (Fig. 5d, e), but greatly downregulated YY1 protein expression by 40% (Fig. 5f). These results implied that PP2A affected YY1 protein expression possibly mainly through YY1 protein stabilization rather than YY1 promoter activity or transcription. Therefore, we confirmed that the ubiquitination of YY1 was expectedly induced by forskolin, compared with that of control groups (Fig. 5g), suggesting that activation of PP2A induced YY1 protein degradation.

### Cisplatin inhibited PPP2CA expression through upregulation of YY1 expression

**Fig. 6:**
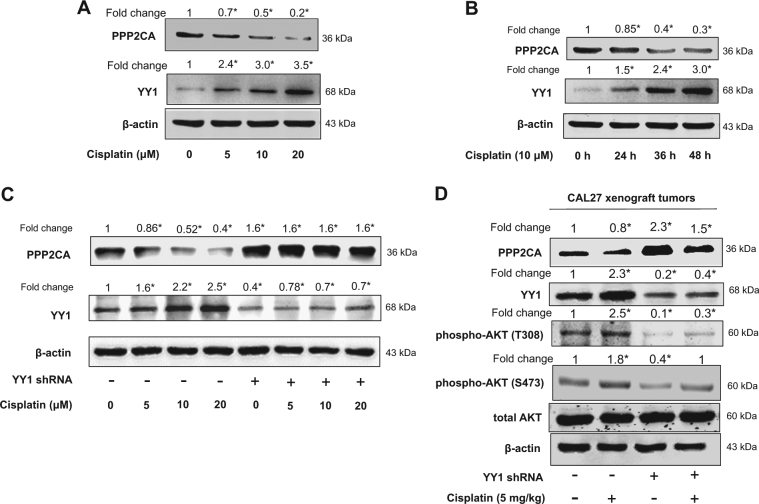
Cisplatin inhibited PPP2CA expression through upregulation of YY1 expression. **a**, **b** Expressions of YY1 and PPP2CA after treatment with cisplatin. CAL27 cells were exposed to different dosage of cisplatin for 48 h (**a**); CAL27 cells were exposed to cisplatin (10 μM) and protein was collected at indicated time points (**b**), total proteins were subjected to Western blot analysis. **c**, **d** Knockdown of YY1 rescued cisplatin-induced PPP2CA protein inhibition. CAL27 cells stably infected with YY1 shRNA or scramble shRNA lentivirus and cells exposed to different dosage of cisplatin (**c**). Proteins were extracted from xenograft tumors (**d**) and total proteins were subjected to Western blot analysis. **a**–**d** The target bands were exposed, and densitometry was performed as fold change ratio from the mean of three independent experiments, one-way ANOVA: *P < 0.05 vs. control group

Cisplatin upregulated YY1 protein expression and simultaneously inhibited PPP2CA protein expression in a time- and dose-dependent manner (Fig. 6a, b). We then confirmed that cisplatin-induced inhibition of PPP2CA protein expression was dependent on its upregulation of YY1 protein expression. As shown in Fig. 6c, in the presence of cisplatin, knockdown of YY1 totally blocked cisplatin-induced inhibition of PPP2CA protein expression and still upregulated PPP2CA protein expression as compared with that of the control. Similar results were observed in xenograft tumors (Fig. 6d).

## Discussion

In present study, we showed for the first time that knockdown of YY1 sensitized HNSCC cells to cisplatin through PP2A/AKT signaling pathway.

Knockdown of YY1 synergistically enhanced cisplatin-induced anticancer effects in HNSCC cell lines. Knockdown of YY1 either by tetracycline-inducible shRNA system or by lentivirus-mediated shRNA system could both synergistically enhance cisplatin-induced apoptosis and inhibition of proliferation, migration and invasion. These effects of YY1 knockdown were also confirmed in the xenograft tumor growth in nude mice. These results strongly suggested that knockdown of YY1 would be beneficial in cisplatin chemotherapy. Considering the results in the present study and previous studies, in which knockdown of YY1 by siRNA sensitizes PC-3 cell to tumor necrosis factor-related apoptosis-inducing ligand (TRAIL)-mediated apoptosis^36–39^, and inhibition of YY1 expression plays an important role in rituximab or galiximab to sensitize B-NHL cell lines to Fas-induced apoptosis or TRAIL-induced apoptosis, respectively^37,39^, it appeared that YY1 was broadly involved in resistance of chemotherapy by cisplatin or other agents. In addition, we also observed that YY1 mRNA expression was more highly expressed in tongue cancer than in the adjacent normal tissue. It would be necessary to explore in the future study whether or not the highly expressed YY1 in the tongue cancer would contribute to resistance of chemotherapy by cisplatin or other anticancer agents. Considering that cisplatin is still one of first-line drugs in treatment of HNSCC^56,57^ the potential clinical significance of our results would be that knockdown of YY1 might increase efficiency of cisplatin or decrease the amount of cisplatin in treatment of HNSCC. Especially in cisplatin resistant HNSCC, knockdown of YY1 might also provide an opportunity to reverse the resistance.

Knockdown of YY1 enhanced cisplatin-induced inhibition of cell proliferation through dephosphorylating T308 of AKT. Acquired AKT phosphorylation or activation is a main reason for cisplatin resistance^9,58^. Knockdown of YY1 inhibited phosphorylation of both S473 and T308 of AKT, whereas overexpression of YY1 upregulated phosphorylation of both S473 and T308, in three HNSCC cell lines examined. These results indicated that YY1 was an important regulator of AKT phosphorylation or activation. Although cisplatin could simultaneously upregulate YY1 protein expression and AKT phosphorylation (S473 and T308) in a time- and dose-dependent manner, knockdown of YY1 blocked cisplatin-induced AKT phosphorylation (S473 and T308) and correspondingly sensitized CAL27 cells to cisplatin in vitro and their xenograft tumor to cisplatin in vivo. These results suggested that cisplatin-induced phosphorylation or activation of AKT was dependent on its upregulation of YY1 expression. Although T308 phosphorylation is necessary and sufficient for AKT activation^18,20,21^, YY1 regulated AKT phosphorylation at both S473 and T308 in our study. We then confirmed by series experiments that T308 dephosphorylation was responsible for YY1 knockdown-induced enhancement of anti-proliferative effect of cisplatin. The most important experiment was that PP2A antagonist, okadaic acid, rescued YY1 knockdown-induced downregulation of T308, but not S473, phosphorylation, and correspondingly abolished YY1 knockdown-induced inhibition of cell proliferation. These results also suggested YY1/AKT signaling pathway contributed to cisplatin resistance. However, other mechanisms may also involve in YY1 knockdown-mediated enhancement of anticancer agents. For example, rituximab inhibited the expression of YY1 resulting in the upregulation of Fas expression and sensitization of the tumor cells to CH-11 (FasL agonist mAb)-induced apoptosis^37^. Inactivation of endogenous YY1 enhances the accumulation and activation of p53 as well as the expression of p53 target genes to sensitize U2OS cells to DNA damage induced apoptosis^59^ and also HeLa cells to As2O3 induced apoptosis^34^. Novel proteasome inhibitor NPI-0052 inhibited the expression of YY1 resulting in upregulation of Death Receptor 5 (DR5) expression and sensitization of PC-3 and B-NHL cells to TRAIL-induced apoptosis^60^. Knockdown of YY1 resulting in the inhibition of multi-drug resistance (MDR1) expression and sensitization of the PC-3 cells to adriamycin-induced apoptosis^61^. Therefore, the mechanisms of YY1 knockdown enhancing chemotherapeutic effects seem to be dependent on different anticancer agents.

Cisplatin could upregulate both YY1 promoter activity and YY1 mRNA stabilization to contribute to the upregulation of YY1 protein expression. Although the detailed mechanism underlying cisplatin-mediated stabilization of YY1 mRNA remains to be fully explored, we had preliminarily observed that it was related to 3′-UTR of YY1 mRNA. This is similar to the results of a previous study, in which cisplatin also increases TNF-α mRNA stability possibly through the binding of certain proteins to AU region in the 3′-UTR^62^. Future studies are needed to elucidate the mechanisms underlying cisplatin-mediated increase in promoter activity and mRNA stabilization of YY1.

PP2A involved in YY1/AKT signaling pathway in cisplatin resistance. We confirmed that YY1 as a transcription factor could regulate AKT phosphorylation (activation) through PP2A, an important phosphatase for regulation of AKT phosphorylation at T308. The evidence was stated as follows. First, overexpression of YY1 partially rescued AKT (T308) phosphorylation after inhibition of PI3K by its specific inhibitor, LY294002, suggesting that overexpression of YY1 somehow inhibited the process of dephosphorylating AKT. Second, overexpression of YY1 downregulated phosphatase PP2A activity and its catalytic subunit alpha PPP2CA protein expression, and correspondingly upregulated AKT phosphorylation at T308, whereas knockdown of YY1 upregulated phosphatase PP2A activity and PPP2CA protein expression, and correspondingly downregulated AKT phosphorylation at T308. Third, more importantly, PP2A antagonist okadaic acid or PPP2CA siRNA both blocked YY1 knockdown-mediated upregulation PPP2CA protein expression, and correspondingly rescued YY1 knockdown-mediated downregulation of AKT phosphorylation at T308. Forth, PP2A agonist, forskolin, rescued YY1 overexpression-mediated inhibition of PPP2CA protein expression, and correspondingly blocked YY1 overexpression-mediated induction of AKT phosphorylation at T308. Fifth, YY1 affected PPP2CA mRNA and protein expressions mainly depending on its binding site A (−1368/−1363) in the PPP2CA promoter. YY1 can either activate or repress gene transcription, depending on its associated proteins and the cell types and situations^24,63–65^. In the present study, YY1 acted again as a repressor for PPP2CA transcription. Our results that YY1 was a negative regulator of PP2A activity through repressing PPP2CA transcription was similar to that of a previous study, in which the transcription factor Ikaros also negatively regulates PP2A activity through repressing PPP2CA transcription by recruiting histone deacetylase 1 (HDAC1)^51^. Whether YY1 also recruits HDAC1 to repress PPP2CA transcription remains to be determined in future. The identification of YY1-binding site A responsible for YY1-mediated inhibition of PPP2CA expression might be a target for future development of anticancer drugs.

**Fig. 7:**
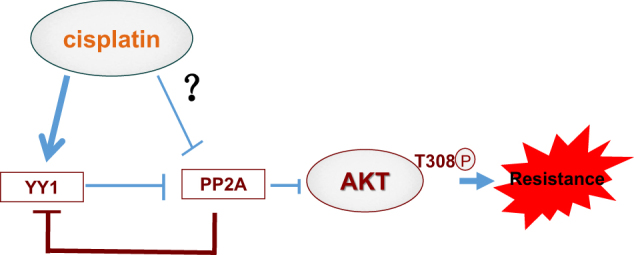
A schematic model of YY1-promoted AKT phosphorylation and involvement in cisplatin resistance. Cisplatin induces YY1 expression, and then upregulated YY1 inhibits PP2A activity and upregulates AKT phosphorylation (mainly T308). Meanwhile, PP2A inactivation conversely further upregulates YY1 expression and promotes YY1-induced AKT activation. Acquired AKT activation confers cancer cells resistance to cisplatin

Interestingly, upregulation of PP2A activity also downregulated YY1 expression through promoting YY1 protein degradation. We observed that activation of PP2A by forskolin enhanced the poly-ubiquitination or degradation of YY1 protein. Ectopical expression of YY1 represses the transcription of endogenous YY1 gene, implying that YY1 negatively auto-regulates the transcription of itself^66^. However, our results showed that ectopical expression of YY1 induced endogenous YY1 protein expression and simultaneously inhibited PPP2CA expression, and that PP2A agonist forskolin downregulated endogenous YY1 protein expression and even blocked ectopical YY1 expression-induced upregulation of endogenous YY1 protein expression. Our results suggested that at protein level, YY1 was positively auto-regulated, although at transcriptional level it was negatively auto-regulated^66^. It was PP2A that mediated the positively auto-regulation of YY1 protein expression, since overexpression of YY1 downregulated PP2A activity, which would result in less degradation of endogenous YY1 protein. According to our results, we speculated that cisplatin would likely first mainly upregulate YY1 expression, then trigger YY1/PP2A/AKT signaling pathway and subsequently have cells to be resistant to cisplatin (Fig. 7).

In conclusion, T308 dephosphorylation of AKT was responsible for YY1 knockdown-induced enhancement of anticancer effects of cisplatin. Targeting on YY1 or PP2A or AKT would help reverse cisplatin resistance in HNSCC.

## Materials and methods

### Cell lines

Human tongue squamous cell carcinoma derived CAL27 cells, human embryonic kidney derived 293T cells, human HNSCC-derived WSU-HN6 cells were maintained in Dulbecco’s modified Eagle’ medium (GIBCO, Grand Island, NY, USA) with 10% fetal bovine serum (FBS) at 37 °C under 5% CO_2_. Human oral squamous cell carcinoma derived SCC9 cells were maintained in a mixture of Dulbecco’s Modified Eagle’s medium and Ham’s F12 medium (1:1) (Invitrogen, Burlington, Ontario, Canada) supplemented with 10% fetal bovine serum (FBS, Invitrogen), 400 micrograms per litre (mg/L) hydrocortisone (Sigma-Aldrich, St Louis, MO, USA). CAL27, 293T, WSU-HN6 and SCC9 cells were obtained from the American Type Culture Collection (ATCC, Manassas, VA, USA). Since cisplatin could induce severe cell death leading to cells floating, in which some molecules such as phosphorylated AKT might be undetectable (Supplemental Figure 11), all the molecular assays such as Western blot and real-time polymerase chain reaction (PCR) were done only from the attached cells.

### Reagents and antibodies

Forskolin, perifosine, BX-795, and cisplatin were purchased from Selleck Chemicals (Houston, TX, USA). Okadaic acid and doxycycline were purchased from Sigma-Aldrich (St. Louis, MO, USA). Antibodies for β-actin and HA-Tag were purchased from Santa Cruz Biotechnology (Santa Cruz, CA, USA). Antibodies for YY1 were purchased from Santa Cruz Biotechnology, H-10, SC-7341 (Santa Cruz, CA, USA) or Cell Signaling Technology, #2185 (Danvers, MA, USA). Antibody for PPP2CA was purchased from EMD Millipore (Merck KGaA, Darmstadt, Germany). Antibodies for phospho-AKT (T308), phospho-AKT (S473), pan-AKT, Flag**-**Tag, and green fluorescent protein (GFP) were purchased from Cell Signaling Technology (Danvers, MA, USA).

### Expression constructs

Full-length of YY1 (NG_046908.1) and AKT (NG_046997.1) coding sequence were amplified from cDNA of CAL27 with a high-fidelity DNA polymerase KOD FX (TOYOBO, Osaka, Japan) by using standard PCR techniques. The PCR products were cloned into pZeroBack/blunt vectors (Tiangen, Beijing, China). YY1 was re-cloned into pEGFP-C1 plasmids at KpnI and XmaI sites and pLVX-AcGFP-N1 vectors (Clontech, Mountain View, CA, USA) at EcoRI and SmaI sites, respectively. AKT was re-cloned into pLVX-AcGFP-N1 vectors (Clontech, Mountain View, CA, USA) at EcoRI and BamHI sites, and according to reported early^67^, we then constructed constitutively active AKT (CA-AKT) by mutation of both serine at 473 site and threonine at 308 site into aspartic acid with following sequence, S473D: TCCCCCAGTTC*GAC*TACTCGGCCAG (sense); CTGGCCGAGTA*GTC*GAACTGGGGGA (antisense); T308D: GCCACCATGAAG*GAC*TTTTGCGGCACA (sense); TGTGCCGCAAAA*GTC*CTTCATGGTGGC (antisense). Italic refer to mutation nucleotides. All the constructs were confirmed by DNA sequencing. ALL plasmids were transfected by Mirus (Mirus Bio, WI, USA). HA-ubiquitin plasmid was kindly provided by Professor Shiaw-Yih Lin at department of Systems Biology, University of Texas M. D. Anderson Cancer Center, Houst YY1 5′-UTR on, USA^68^.

### 5′-UTR and 3′-UTR luciferase constructs

Nucleotide sequences of YY1 5′-UTR and 3′-UTR were acquired from the GenBank (NM_003403.4). We designated the translation start site of YY1 as +1, therefore, the downstream of the coding sequence (+1246 to +2679) was 3′-UTR, and the upstream of the coding sequence (−480 to −1) as YY1 5′-UTR that was consistent with a previous study^32^. YY1 3′-UTR and 5′-UTR were amplified by PCR and cloned into pZeroback/blunt vectors (Tiangen, Beijing, China) and re-cloned into pGL3-Control plasmids (Promega) at XbaI sites, respectively. The primers for cloning the 5′-UTR of YY1 were as follows: TCTAGAAGGGCGAACGGGCGAGTGGC (sense); TCTAGACCATGGCTGAGGGCTCCGCCG (antisense). The primers for cloning the 3′-UTR of YY1 were as follows: TCTAGA AAAGAAGAGAGAAGACCCTTCTCGACC (sense); TCTAGA AGAAACATGAAATTAAGCTACTGGCACTCA (antisense). All the constructs were confirmed by DNA sequencing.

### Small interfering RNAs and short hairpin RNA

Small interfering RNAs (siRNA) of human YY1, PPP2CA (NG_012188.1) and AKT were purchased from Santa Cruz Biotechnology (Santa Cruz, CA, USA) or commercially synthesized based on the following sequence, YY1 siRNA: 5′- GAACUCACCUCCUGAUUAU-3′ (sense); 5′- AUAAUCAGGAGGUGAGUUC-3′ (antisense); PPP2CA siRNA: 5′- GGAUAGCAGCAAACAAUCAUUGGAG-3′ (sense); 5′-CUCCAAUGAUUGUUUGCUGCUAUCC-3′ (antisense). siRNAs were transfected by Lipofectamine 3000 (Invitrogen, Carlsbad, CA, USA). Short hairpin RNA (shRNA) for human YY1 was commercially synthesized based on the following sequence (YY1 shRNA-1): 5′- ACCGGGGGAGCAGAAGCAGGTGCAGATCTCGAGATCTGCACCTGCTTCTGCTCCCTTTTTGAATTC-3′ (sense); 5′- GAATTCAAAAAGGGAGCAGAAGCAGGTGCAGATCTCGAGATCTGCACCTGCTTCTGCTCCCCCGGT-3′ (antisense)^69^, and then cloned into PLKO-Tet-on plasmids (Addgene Cambridge, MA, USA) at AgeI and EcoRI sites that was the “all-in-one” system for the inducible expression of shRNA. Human YY1 shRNA-2 was commercially synthesized as follow sequence: 5′- GATCCCCGGCAGAATTTGCTAGAATGTTCAAGAGACATTCTAGCAAATTCTGCCTTTTTA -3′ (sense); 5′- AATTCAAAAAGGCAGAATTTGCTAGAATGTCTCTTGAACATTCTAGCAAATTCTGCCGGG -3′ (antisense)^70^ and then cloned into PLVX-shRNA1 vectors (Clontech, Mountain View, CA, USA) at BamHI and EcoRI sites. All constructs were confirmed by DNA sequencing.

### Stable transfection with lentivirus

The plasmids pLVX-AcGFP-N1, pLVX-AcGFP-N1-YY1, pLVX-AcGFP-N1-CA-AKT, PLKO-Tet-on-shYY1-1 and PLVX-shYY1-2 were each co-transfected into 293T cells by using PLP1, PLP2 and VSVG lentiviral packing plasmids (Clontech). Lentiviral supernatants were collected 48 h (h) after transfection and then centrifuged (500 g for 10 min at 4 °C). The supernatant was added to CAL27 cells. The infected CAL27 cells were screened using 1 microgram per milliliter (μg/ml) puromycin. Overexpression or knockdown of YY1 in CAL27 cells was confirmed with Western blot assay.

### Protein extraction and Western blot analysis

The attached cells were collected after wash with ice-cold PBS gently for three times. Cell lysates were extracted using RIPA lysis buffer (Solarbio, Beijing, China). Protein concentrations were determined by BCA protein assay (Thermo Fisher Scientific Inc). Equal amounts of samples were subjected to 8–10% sodium dodecyl sulfate-polyacrylamide gel electrophoresis and transferred to a nitrocellulose filter membrane (Millipore, Billerica, MA, USA). The membrane was blocked with 5% non-fat milk in TBS-T (50 mmol/L Tris, pH 7.5; 150 mmol/L NaCl; 0.05% Tween 20) for 1 h at room temperature (RT). The membrane was incubated with anti-phosphorylated protein antibody at 1:1000 in TBS-T overnight at 4 ℃, washed with TBS-T three times and then incubated with secondary horseradish peroxidase-conjugated antibody for 1 h at RT. After extensive washes with TBS-T, the membrane was visualized with enhanced chemiluminescence plus reagents (Thermo) by FUSION FX imaging system (VILBER, France). The membrane was stripped for detection of YY1 or PPP2CA with anti-YY1 or PPP2CA antibodies, respectively, and finally the membrane was tripped for detection of β-actin. The densitometry of target band was assessed by the Image-J software. Data were presented as mean ± standard deviation (SD) of at least three independent experiments.

### Real-time quantitative PCR

Total RNA was extracted using TRIzol reagent (Invitrogen, Carlsbad, CA, USA). Reverse transcription and real-time PCR were performed as described previously^71^. The primers for human YY1 gene as follows: 5′- GACCTCTCAGATCCCAAA-3′ (sense) and 5′- TTGTTTTTGGCCTTAGCA-3′ (antisense). The primers used for human *PPP2CA* gene as follows: 5′-GATCTTCTGTCTACATGGTGGTCTC-3′ (sense) and 5′-ACACATTGGACCCTCATGGGGAA-3′(antisense). The primers for human YY1 and human PPP2CA were designed using the Primer Premier version 5.0 software (Premier, Canada). The primers used for human β-actin were: 5′-CGGGAAATCGTGCGTGAC-3′ (sense) and 5′-CAGGCAGCTCGTAGCTCTT-3′ (antisense). All the primers were commercially synthesized and the efficiency of all the primers was confirmed by sequencing their conventional PCR products. Real-time PCR was performed as described previously using a 7500 real-time PCR system of Applied Biosystems (Invitrogen, Carlsbad, CA, USA) with FastStart Universal SYBR Green Master Roche (Basel, Swiss) according to manufacturer instruction.

### PP2A immunoprecipitation phosphatase assays

The phosphatase activity of PP2A was measured in the protein extracts with a PP2A immunoprecipitation phosphatase assay kit from Millipore (Merck KGaA, Darmstadt, Germany) following the manufacturer’s instructions. Briefly, PPP2CA was immunoprecipitated using 4 micrograms (μg) of antibody for PPP2CA and 25 microlitre (μl) Protein A agarose slurry for 16 h in constant rocking at 4 ℃. The samples were washed 3 times with TBS and then followed by one additional wash with Ser/Thr assay buffer. Next, 60 μl of a diluted phosphopeptide at 750 μM and 20 μl of Ser/Thr assay buffer were added, and the mix was incubated for 10 minutes (min) at 30 ℃ in a shaking incubator, and then 25 μl of the mix was transferred into each well of a 96-well plate and 100 μl of Malachite Green Detection Solution was added, and the mix was incubated for 15 min at RT. Absorbance at 650 nm was used to calculate the amount of phosphate released using a standard curve (0–2000 pmol). Each measurement was performed in triplicates.

### Promoter reporter constructs

The sequence of the human PPP2CA promoter and YY1 promoter were obtained from GenBank. The putative full-length promoter of human PPP2CA (−2000 to +88) and of YY1 (−1500 to +40) was amplified from the genomic DNA of CAL27 cells with a high-fidelity DNA polymerase KOD FX (TOYOBO) using standard PCR techniques. The translational start site was identified as +1. The PCR products were cloned into pZeroback/blunt vectors (Tiangen, Beijing, China) and YY1 promoter re-cloned into pGL3-Basic plasmids (Promega) at KpnI and HindIII sites, whereas PPP2CA promoter re-cloned into pGL3-Basic plasmids (Promega) at KpnI and NheI sites. The constructs were confirmed by DNA sequencing.

### Site-directed mutagenesis

Site-directed mutagenesis was performed via PCR with a high-fidelity DNA polymerase KOD FX (TOYOBO) as described previously^71^. All primers were custom synthesized (Sangon Biotech Co., Shanghai, China). The primers used for deletion of YY1-binding site A (−1464/−1363) on the PPP2CA promoter were as follows: 5′-TCTCTCTTCTGCCTATCC*CAAACCATCTTC*AGCCAGTACTGTTGGGAG-3′ (sense); 5′- TCTCCCAACAGTACTGGCTGGAT*GAAGATGGTTTG*AGGCAGAAGAGAGAT-3′ (antisense); the primers for deletion of YY1-binding site B (−1195/−1066) on the PPP2CA promoter as follows: 5′-ACGCGGTCAGGACAGGT*GCATCCATCTTC*TCTGGGCTTCCCCTCTTGAAA-3′ (sense); 5′-TTTCAAGAGGGGAAGCCCAGA*GAAGATGGATGC*ACCTGTCCTGACCGCGT-3′ (antisense). In the above primers, the italics refer to the nucleotides deleted. All constructs were confirmed by DNA sequencing.

### Luciferase assay

Luciferase assay was performed as described previously^72^. Briefly, 1 μg of plasmids was transfected with Mirus (Mirus Bio, WI, USA) into the CAL27-vector cells, CAL27-shYY1-2 cells, Cal27-Dox-indu-shYY1-1 cells, and CAL27-PLVX-GFP-YY1 cells, respectively, and cultured in a 12-well plate. The CAL27-Dox-indu-shYY1-1 cells was induced using doxycycline (100 mg/L) 48 h before transfection. Eight hours after transfection, cisplatin was added into the medium. The transfected cells were lysed with cell lysis buffer (Promega, Fitchburg, WI, USA) 28 h after transfection. Luciferase activities were measured with LB960 microplate luminometer (Berthold, Berlin, Germany) using luciferin as the substrate, according to the manufacturer’s instructions (Promega, Fitchburg, WI, USA).

### Immunoprecipitation

Five-hundred micrograms (mg) of whole cell extracts were incubated in 500 µl extraction buffer with 4 micrograms (µg) PPP2CA antibody for 16 h (h) at 4 °C, and then added with 40 µl protein A/G-agarose beads (Santa Cruz Biotechnology) and incubated again for 2 h at 4 °C. After five times wash, the bound proteins were released by boiling in the loading buffer and then subjected to western blot analysis.

### DNA-affinity purification assay

DNA-affinity purification assay (DAPA) was performed as described previously^73^. Briefly, 5′-end-biotinylated oligonucleotides were custom synthesized with the sequence corresponding to the PPP2CA promoter −1371/−1358 region containing the putative YY1 binding consensus (the italic) designated as A site (5′-biotin/ CCCAA*ACCATC*TTCAG-3′) and the PPP2CA promoter −1100/−1063 region containing the putative YY1 binding consensus (the italics) designated as B site (5′-biotin/GGT*GCATCCATCTTC*TCT-3′). The biotinylated sense strand was annealed with its non-labeled antisense strand and then incubated with CAL27 nuclear extracts. The streptavidin-agarose beads (Sigma-Aldrich) were used to precipitate protein DNA complex. The bound proteins were released by boiling in sodium dodecyl sulfate loading buffer and then subjected to western blot analysis.

### Chromatin immunoprecipitation assay

Chromatin immunoprecipitation (ChIP) assay was performed using a ChIP assay kit (Millipore, Billerica, MA). Briefly, CAL27 cells were cross-linked with 1% formaldehyde when cell density reached about 90% confluence. The chromatin was sonicated into fragments ranging between 200 and 800 bp and then was precipitated by anti-YY1, anti-H3, and anti-IgG antibodies, respectively, for real-time quantitative PCR or conventional PCR. The primers for amplifying the region (−1464/−1363) containing YY1-binding site A of PP2A promoter were as follows: 5′- GCTCTTGCCTTGCCCTTTATG-3′ (sense) and 5′- TCCAGACGGACCAGGGACCAT-3′ (antisense). The primers for amplifying the region (−1195/−1066) containing YY1-binding site B of PP2A promoter were as follows: 5′- TAACCAGACAGAGGTCCAATC-3′ (sense) and 5′- GGTTGCTTTCCTCCAAGTTTA -3′ (antisense). Real-time PCR was preformed using a 7500 real-time PCR system of Applied Biosystems with FastStart Universal SYBR Green Master (Roche). The conventional PCR products were separated on 2% agarose gel.

### Cell proliferation assay

Cell proliferation assay was performed using Cell Counting Kit-8 (CCK-8, Dojindo, Kumamoto, Japan) according to the manufacturer’s instructions. Briefly, the cells were seeded into 96-well plates (1.5 × 10^3^ cells per well) and treated with different reagents. After 48 h treatment, 10 μl of CCK-8 was added to each well containing 100 μl growth medium. After incubation at 37 °C for 3 h, absorbance at 450 nm was determined.

### Assessment of cell apoptosis

Cells were washed with phosphate-buffered saline (PBS) thrice, fixed with 4% paraformaldehyde for 5 min, and incubated with 5 μg/ml 4, 6-diamidino-2-phenylindole dihydrochloride (DAPI) in the dark for 3 min at RT. After washed with PBS, the cells were examined under a fluorescence microscope (Eclipse TS100, Nikon, Japan). Cells presenting features of nuclear condensation and fragmentation were identified as apoptotic cells and were counted within the six randomly selected fields. The rate of apoptotic cells was presented as means ± SD of at least three independent experiments.

### Transwell migration and invasion assay

Cell migration and invasion assays were performed in transwell chambers (Corning Costar, Corning, NY, USA) by using a polycarbonate membrane. Briefly, for migration assays, the cells were seeded at 2 × 10^5^ cells per well in serum-free medium in the upper chambers; the lower chambers contained the growth medium with 10% fetal bovine serum. The cells were then incubated with the reagents in the lower chambers for 16 h. Cells on the top surface of the membrane were wiped off, whereas those on the bottom surface were fixed with 4% paraformaldehyde and stained with 0.01% crystal violet, and examined under a light microscope (BX60, Olympus, Japan), counted and averaged by the number of six randomly selected fields. The same procedure was performed for transwell invasion assay, except that the upper chambers were coated with 20 μg of extracellular matrix gel prior to seeding of the cells (BD, Biosciences).

### Xenograft tumor inoculation

All animal experiments were performed in compliance with the National Institutes of Health guide for the care and use of Laboratory animals. Nude mice (nu/nu, 4 weeks old) were purchased from Beijing Vital River Laboratory Animal Technology Co. Ltd (Beijing, China). The care and treatment of experimental animals followed the institutional guidelines. Mice were randomly allocated to each group (*n* = 5). CAL27-vector cells and CAL27-shYY1 cells were subcutaneously inoculated 2 × 10^6^ cells/mouse or 5 × 10^6^ cells/mouse, respectively, in the left groin of mice. CAL27-Tet-on-shYY1 cells were subcutaneously inoculated (2 × 10^6^ cells/mouse) in the left axilla of mice. After 10 days, nude mice that inoculated CAL27-shYY1 or Cal27-vector cells was intraperitoneal injected cisplatin (5 mg/kg, dissolved in saline) or saline, twice a week for 3 weeks. Nude mice that inoculated CAL27-Tet-on-shYY1 cells was offered water with doxycycline or not and intraperitoneal injected cisplatin or saline, twice a week for 3 weeks. Nude mice was killed, and the weights of xenograft tumors and nude mice were measured.

### Clinical specimens

Clinical specimens of tongue squamous cell carcinoma and adjacent normal tissues were collected as described previously^71^, from 37 patients who underwent surgery in the Department of Oral and Maxillofacial Surgery, Peking University School of Stomatology. Lumps of tumors and adjacent normal tissues, which were at least 1.5 cm distal to the tumor margins, were confirmed by pathological examination. The experiment was approved by the Ethics Committee of Peking University School of Stomatology. Informed consents were obtained from all patients.

### Coefficient of drug interaction

Coefficient of drug interaction (CDI) was performed as described previously^74^. CDI was calculated as follows: CDI = AB/(*A* × *B*). AB is the ratio of the absorbance of the combination treatment group to that of the control group; A or B is the ratio of the absorbance of cisplatin or YY1 knockdown group to that of the control group. Thus, CDI value <1, =1 or >1 indicates that the drugs are synergistic, additive or antagonistic, respectively. CDI less than 0.7 indicates that the drugs are significantly synergistic.

### Statistical analysis

Statistical analysis was performed using SPSS 20 for Windows. All experiments were repeated three times and all data were presented as mean ± SD. Differences between multiple groups were analyzed by one-way analysis of variance (ANOVA); differences between two groups were analyzed by t-test analysis of variance. A value of *P* < 0.05 was considered to be statistical significance.

## Electronic supplementary material

sulpplementary figures
