# Supplementary material for: Knockdown of Yin Yang 1 enhances anticancer effects of cisplatin through protein phosphatase 2A-mediated T308 dephosphorylation of AKT
Source: Cell Death Dis. 2018 Jul 3;9(7):747. doi: 10.1038/s41419-018-0774-8 (PMC6030060; doi:10.1038/s41419-018-0774-8)
Supplement: Supplementary file 1 — sulpplementary figures [file 41419_2018_774_MOESM1_ESM.pdf]

Supplemental Figure 1

A

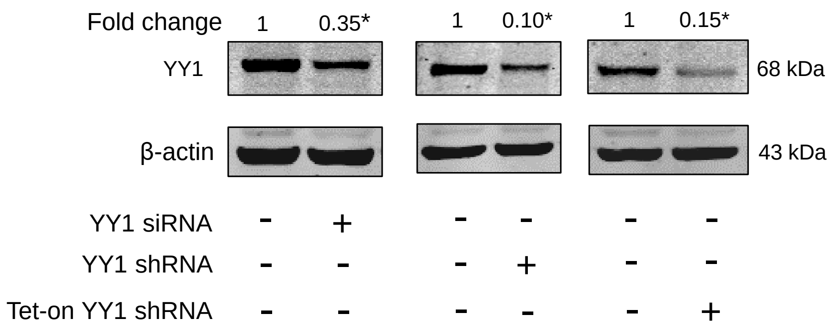

B

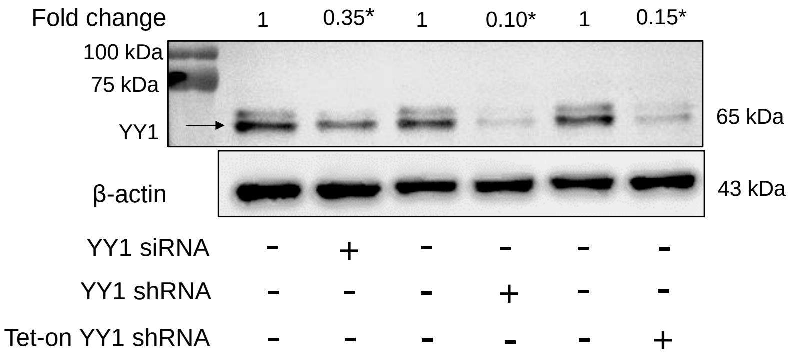

Supplemental Figure 1: Confirmation of efficiency of YY1 knockdown. Cal27 cells transfected with YY1 siRNA for 48 hours or CAL27 cells stably infected with YY1 shRNA or scramble shRNA or CAL27 cells stably infected with Tet-on YY1 shRNA and exposed to doxycycline (100 nM) 48 hours or not. Total protein was extracted and subjected to Western blot analysis. A: YY1 antibody from Santa Cruz; B: YY1 antibody from Cell Signaling Technology. The target bands were exposed, and densitometry was performed as fold change ratio from the mean of three independent experiments. *t*-test, **P* < 0.05 vs. the control group.

Supplemental Figure 2

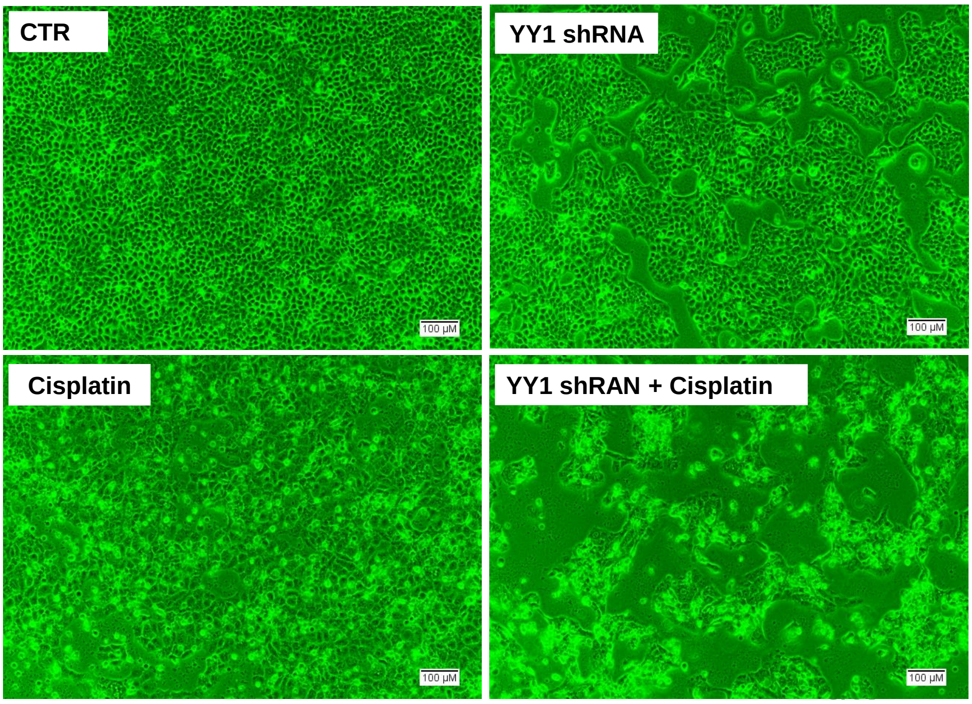

Supplemental Figure 2: Knockdown of YY1 enhanced cisplatin-induced cell proliferation inhibition. Microphotographs of CAL27 cells stably transfected with YY1 shRNA lentivirus were exposed to 10 μM cisplatin or not for 48 h, scale bar: 100 μm.

Supplemental Figure 3

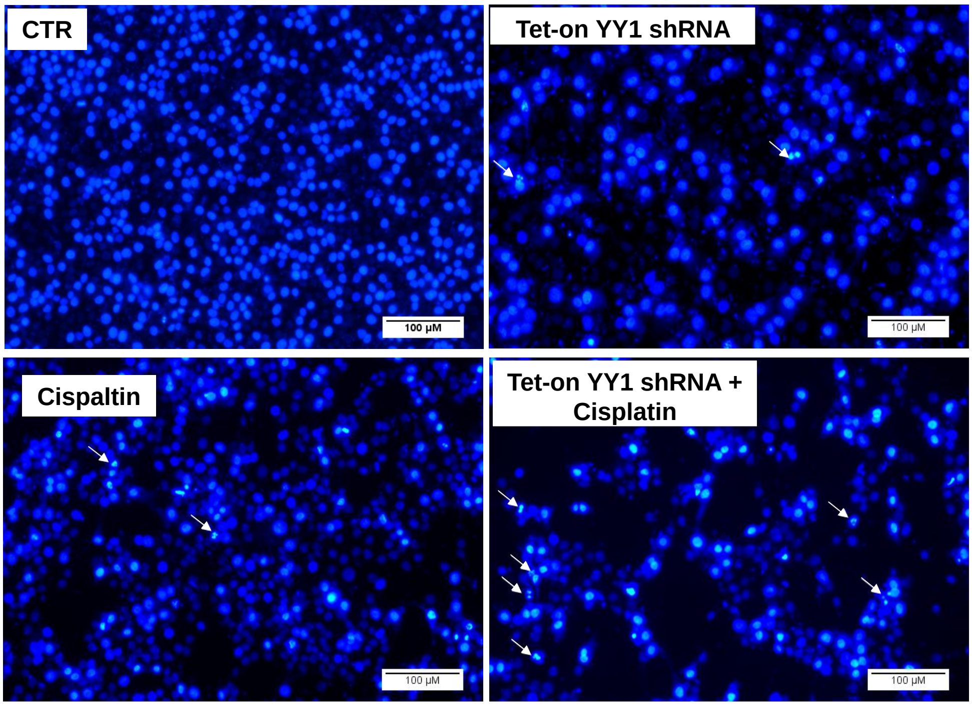

Supplemental Figure 3: Knockdown of YY1 enhanced cisplatin-induced cell apoptosis. Fluorescent microphotographs of CAL27 cells stably transfected with Tet-on YY1 shRNA were exposed to either doxycycline (100 nM) or cisplatin (10 μM) or both for 48 h and subjected to DAPI, scale bar: 100 μm.

Supplemental Figure 4

A B

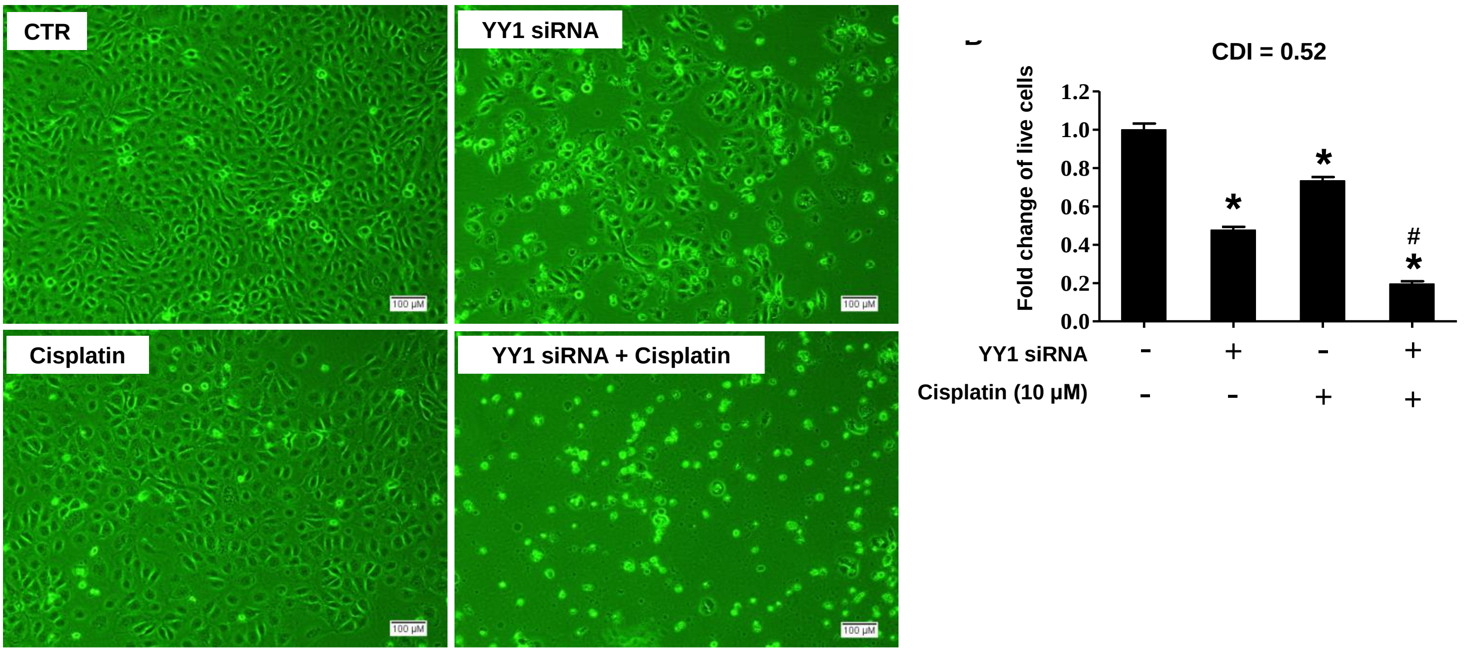

Supplemental Figure 4: Knockdown of YY1 enhanced cisplatin-induced cell proliferation inhibition. SCC9 Cells were transfected with either scramble siRNA or YY1 siRNA and treated with 10 µM cisplatin or not for 48 h. A: microphotographs of SCC9 Cells after different treatment, scale bar: 100 µm. B: SCC9 Cells were subjected to CCK-8 assay. One-way ANOVA: $^{*}P < 0.05$ vs. control group; $^{\#}P < 0.05$ vs. YY1 knockdown or cisplatin group, n = 4.

Supplemental Figure 5

A B

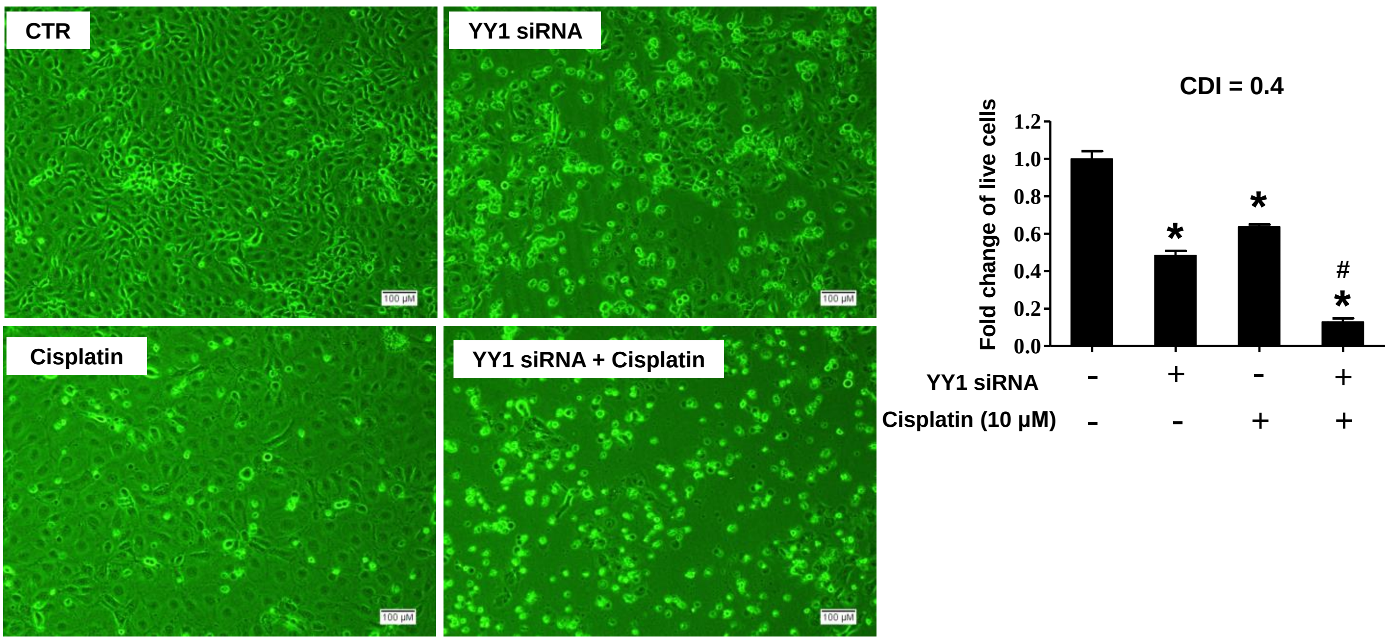

Supplemental Figure 5: Knockdown of YY1 enhanced cisplatin-induced cell proliferation inhibition. WSU-HN6 cells transfected with either scramble siRNA or YY1 siRNA and treated with 10 µM cisplatin or not for 48 h. A: microphotographs of

WSU-HN6 cells after different treatment, scale bar: 100 μm. B: WSU-HN6 Cells were subjected to CCK-8 assay. One-way ANOVA: *P < 0.05 vs. control group; #P < 0.05 vs. YY1 knockdown or cisplatin group, n = 4.

Supplemental Figure 6

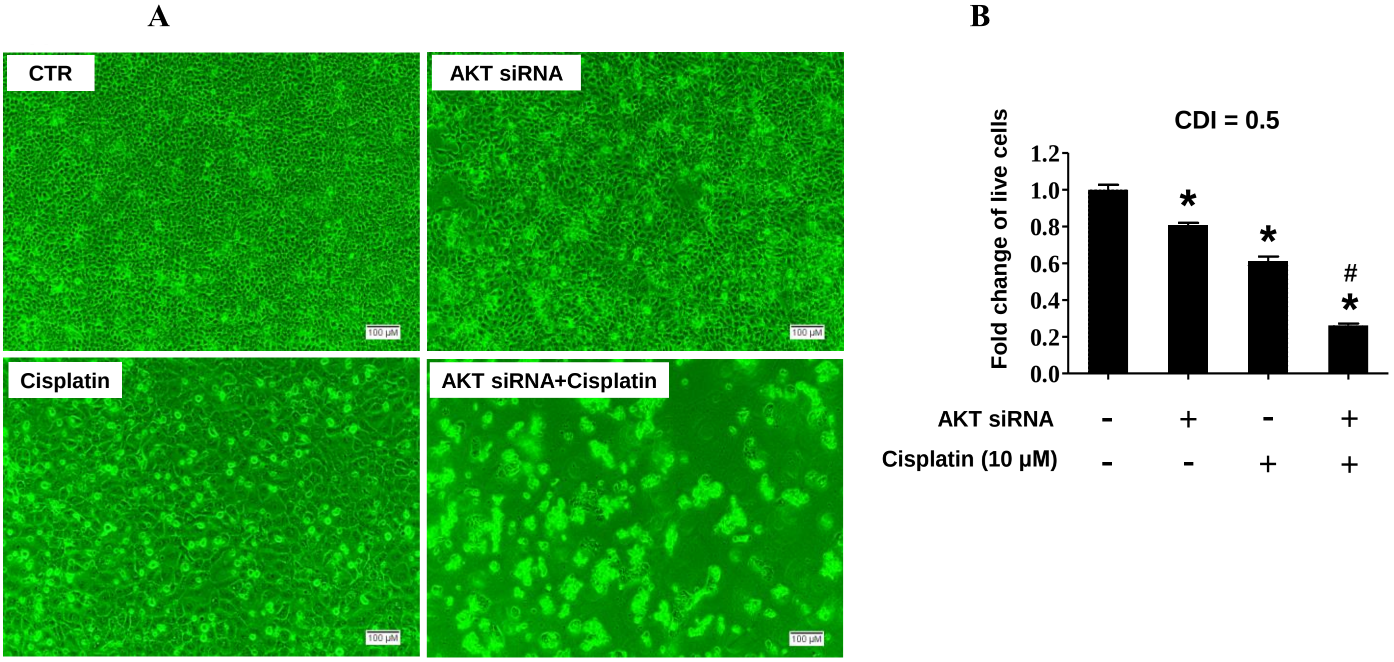

Supplemental Figure 6: Knockdown of AKT enhanced cisplatin-induced cell proliferation inhibition. CAL27 cells transfected with either scramble siRNA or AKT siRNA and treated with 10 μM cisplatin or not for 48 h. A: microphotographs of CAL27 cells after different treatment, scale bar: 100 μm. B: CAL27 Cells were subjected to CCK-8 assay. One-way ANOVA: *P < 0.05 vs. control group; $^{\#}P$ < 0.05 vs. AKT knockdown or cisplatin group, n = 4.

Supplemental Figure 7

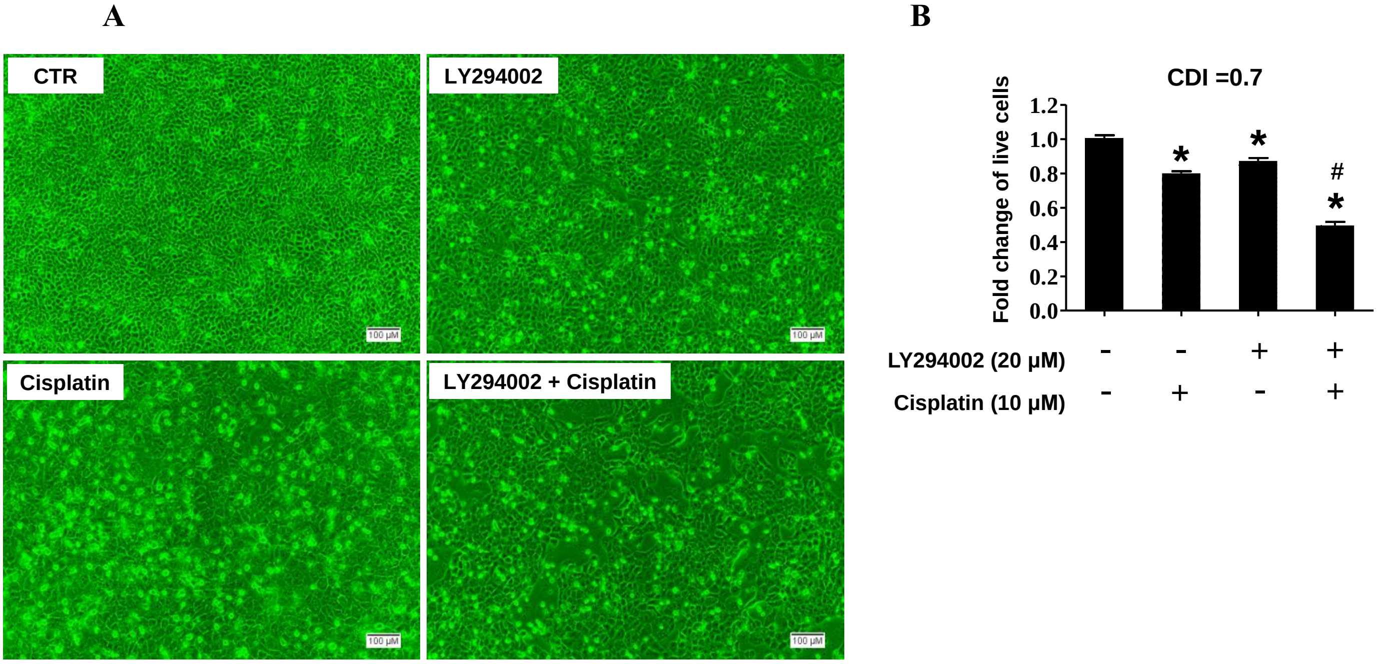

Supplemental Figure 7: PI3K inhibitor (LY294002) enhanced cisplatin-induced cell

proliferation inhibition. CAL27 Cells were treated with either 20μM LY294002 or 10μM cisplatin or both for 48 h. A: microphotographs of CAL27 cells after different treatment, scale bar: 100 μm. B: CAL27 Cells were subjected to CCK-8 assay. One-way ANOVA: $^{*}P < 0.05$ vs. control group; $^{\#}P < 0.05$ vs. LY294002 or cisplatin group, n = 4. (Bars: mean ± SD)

Supplemental Figure 8

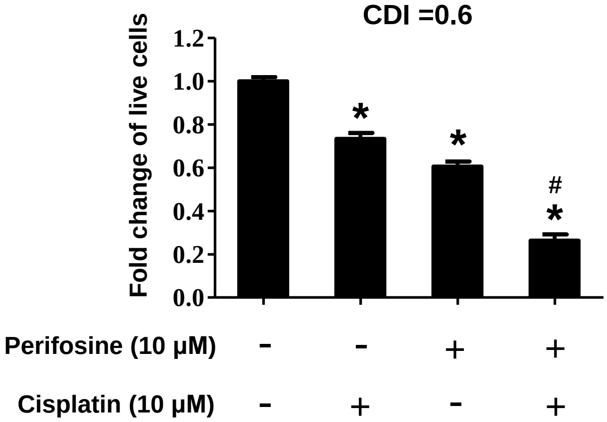

Supplemental Figure 8: Perifosine enhanced cisplatin-induced cell proliferation inhibition. CAL27cells were treated with either 10μM Perifosine or 10μM cisplatin or both for 48 h and subjected to CCK-8 assay. One-way ANOVA: $*P < 0.05$ vs. control group; $\#P < 0.05$ vs. perifosine or cisplatin group, n = 4. (Bars: mean ± SD)

Supplemental Figure 9

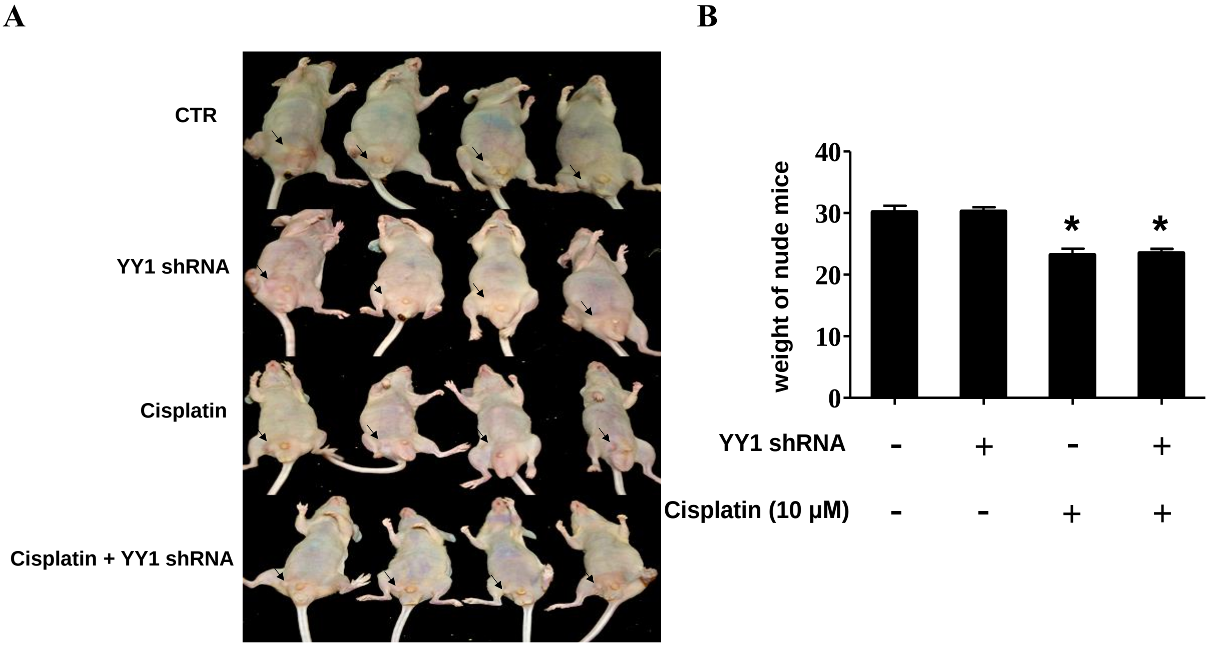

Supplemental Figure 9: nude mice were injected into inguinal region with CAL27 cells which stably transfected with scramble shRNA or YY1 shRNA and intraperitoneal injection with cisplatin or not for 3 weeks. A: pictures of nude mice model after different treatment. B: weight of nude mice model after different treatment. One-way ANOVA:

*$P < 0.05$ (n = 4). (Bars: mean ± SD)

Supplemental Figure 10

A **B**

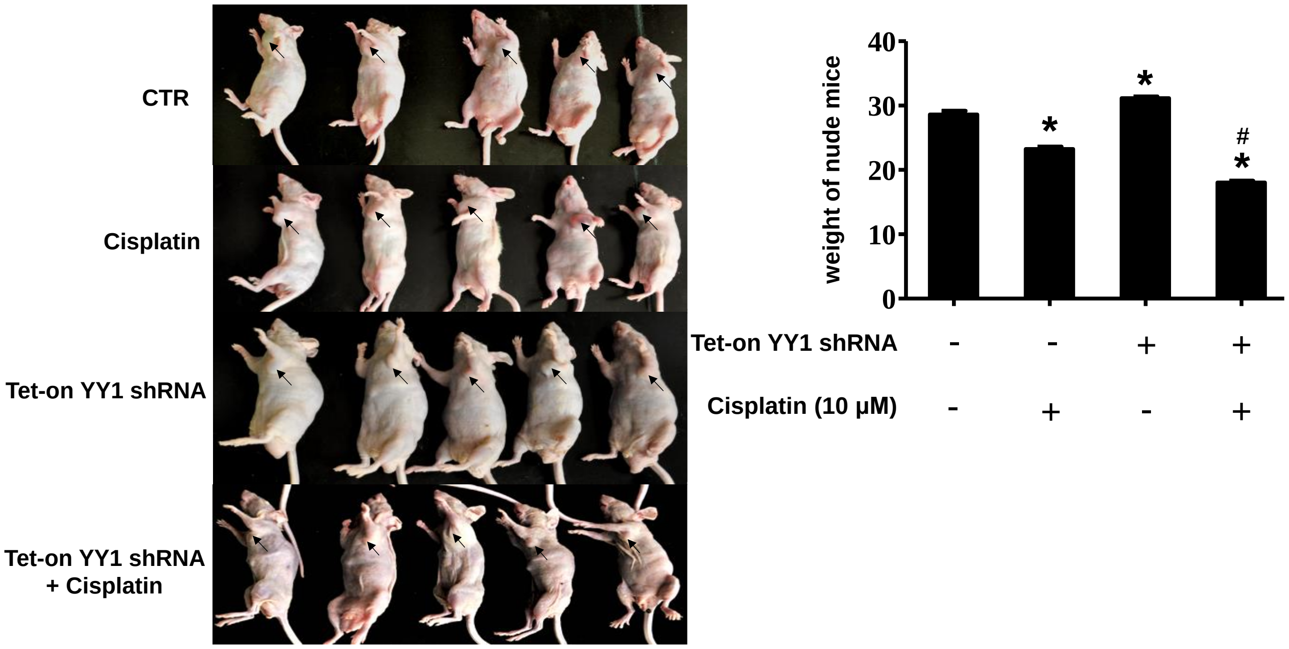

Supplementary Figure 10: nude mice were subaxillary injected with CAL27 cells which stably transfected with Tet-on YY1 shRNA and intraperitoneal injection with cisplatin or feed with doxycycline or both for 3 weeks. A: pictures of nude mice model after different treatment; B: weight of nude mice model after different treatment. One-way ANOVA: *$P < 0.05$ vs. control group; $^{\#}P < 0.05$ vs. YY1 knockdown or cisplatin group, n = 5. (Bars: mean ± SD)

Supplemental Figure 11

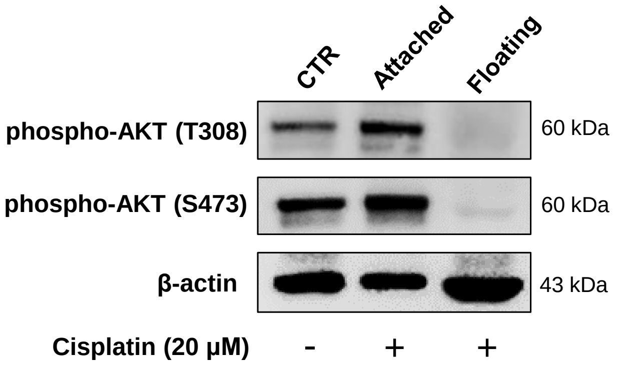

Supplemental Figure 11: AKT phosphorylation at T308 and S473 expression of attached cells and floating cells, respectively. CAL27 cells exposed to 20 μM cisplatin for 48 hours and collected floating cells and attached cells respectively. Protein was extracted and subjected to western blot analysis.

Supplemental Figure 12

A

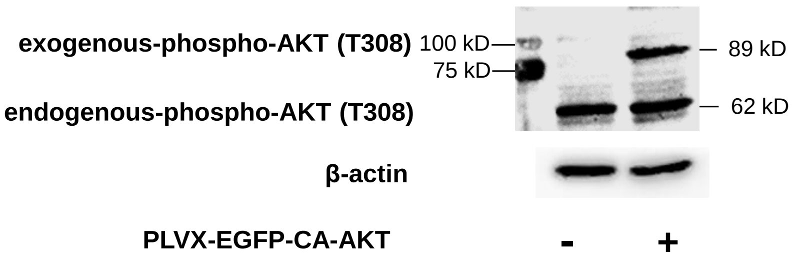

B

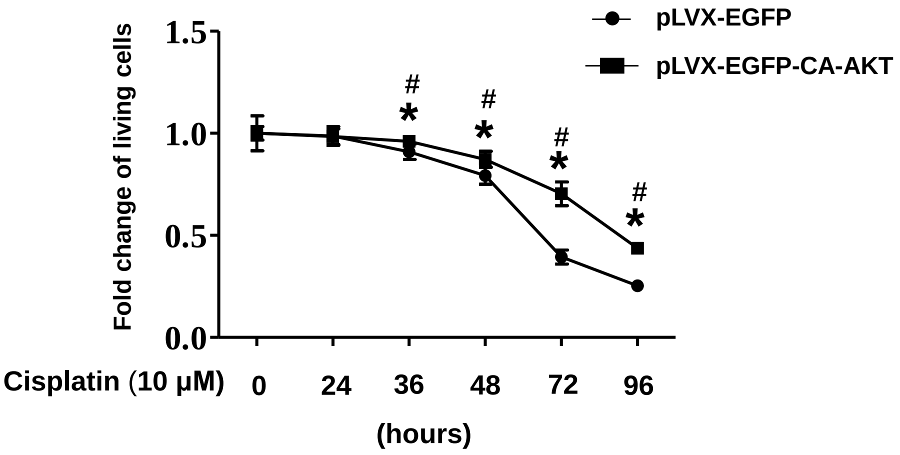

Supplemental Figure 12: A: confirmation the efficiency of constitutively active of AKT. CAL27 cells were stably infected with either constitutively active of AKT (PLVX-EGFP-CA-AKT) lentivirus or GFP empty vector lentivirus and we named them as CAL27-EGFP-CA-AKT cells and CAL27-EGFP cells respectively. Endogenous and exogenous phosphor-AKT (T308) were assessed by western blot. B: CAL27-EGFP-CA-AKT cells were not sensitive for cisplatin-induced proliferation inhibition compared with CAL27-EGFP cells. CAL27-EGFP-CA-AKT cells and CAL27-EGFP cells were treated with 10 μM cisplatin and subjected to CCK-8 assay at indicated times. One-way ANOVA: $^*P < 0.05$ vs. control group. $^{\#}P < 0.05$ vs. CAL27-EGFP-CA-AKT cells (n = 4). (Bars: mean ± SD)

Supplemental Figure 13

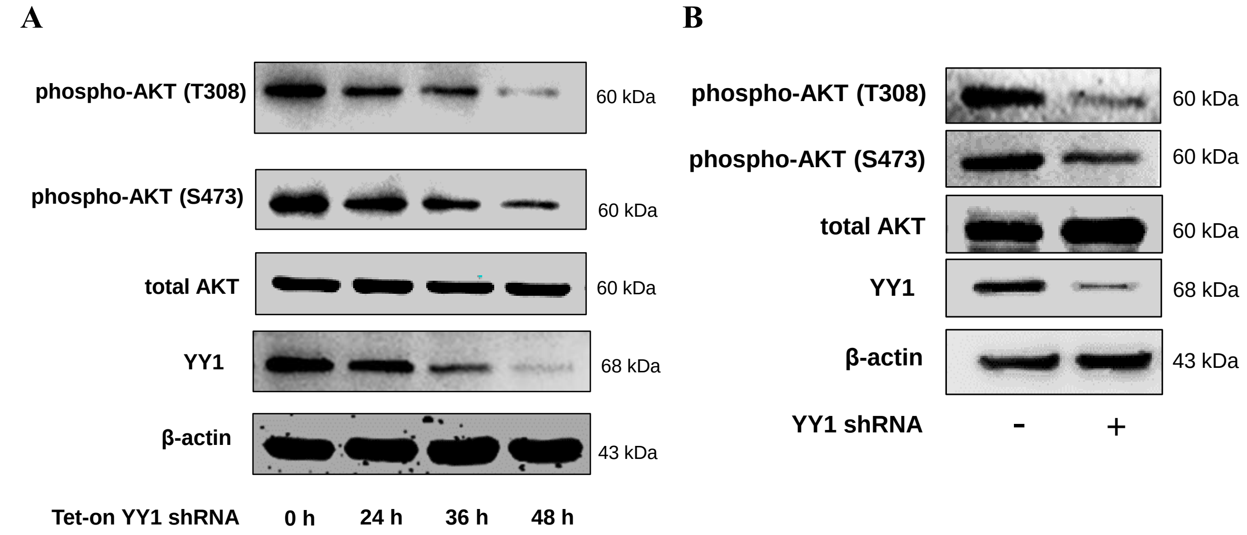

Supplemental Figure 13: Knockdown of YY1 inhibited T308 and S473 phosphorylation of AKT. A: CAL27 cells stably transfected with Tet-on YY1 shRNA were exposed to Doxycycline (100 nM) and collected at indicated time points. B: CAL27 cells were infected with scramble shRNA or YY1 shRNA lentivirus. Cell lysates were analyzed by western blot.

Supplemental Figure 14

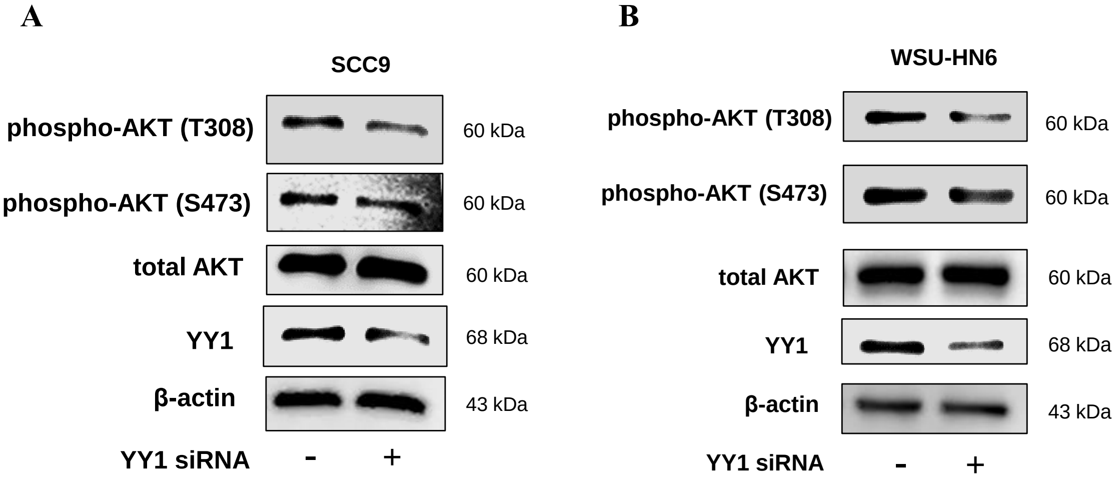

Supplemental Figure 14: Knockdown of YY1 inhibited T308 and S473 phosphorylation of AKT. SCC9 cells (A) and WSU-HN6 cells (B) were transfected with either YY1 siRNA or scramble siRNA for 48 h. Cell lysates were analyzed by western blot.

Supplemental Figure 15

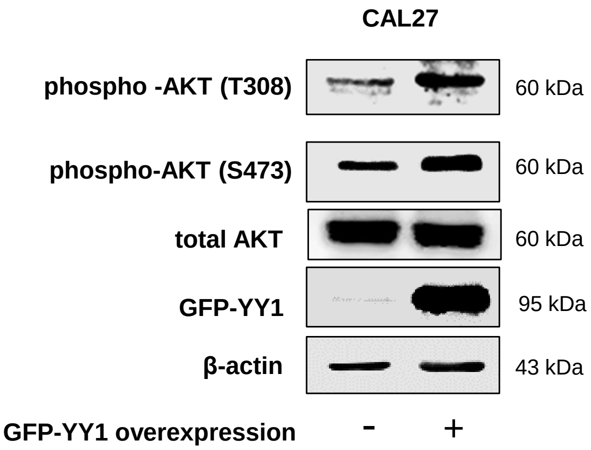

Supplementary Figure 15: YY1 overexpression upregulated T308 and S473 phosphorylation of AKT. CAL27 cells stably transfected with either GFP or GFP-YY1 lentivirus. Cell lysates were analyzed by western blot.

Supplemental Figure 16

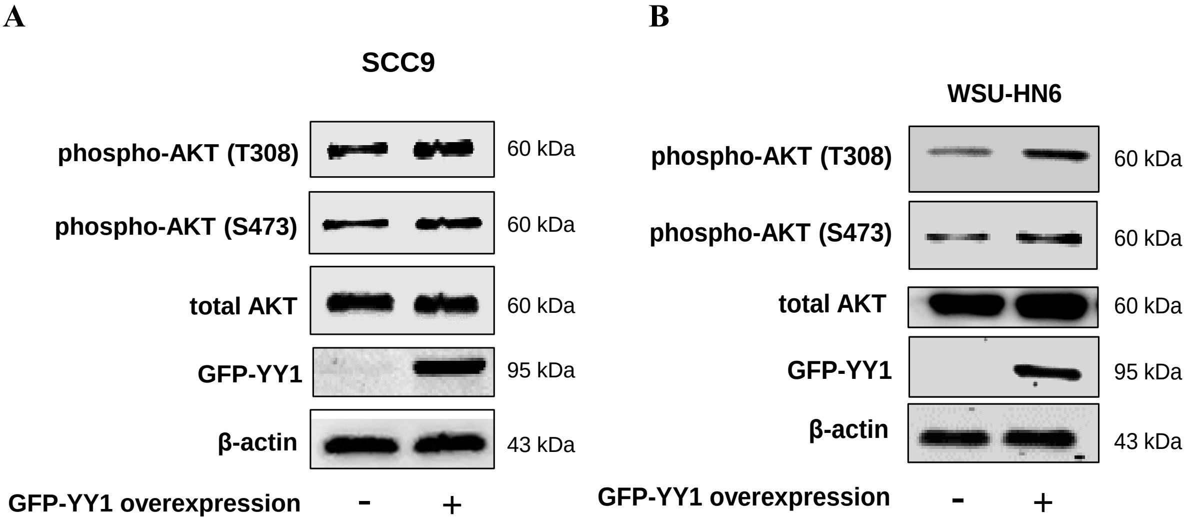

Supplementary Figure 16: YY1 overexpression upregulated T308 and S473 phosphorylation of AKT. SCC9 (A) and WSU-HN6 (B) cells transfected with either GFP or GFP-YY1 vectors for 48 h. Cell lysates were analyzed by western blot.

Supplemental Figure 17

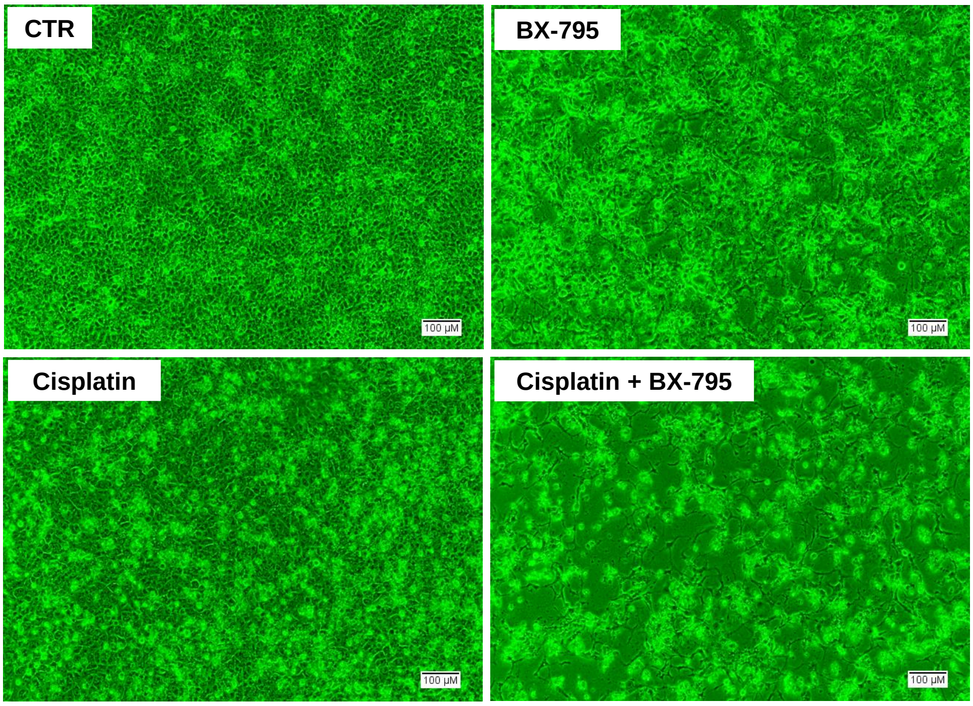

Supplemental Figure 17: PDK1 inhibitor (BX-795) enhanced cisplatin-induced cell proliferation inhibition. Microphotographs of CAL27 cells were treated with BX-795 (10 μM) or cisplatin (10 μM) or both for 48 h, scale bar: 100 μm.

Supplemental Figure 18

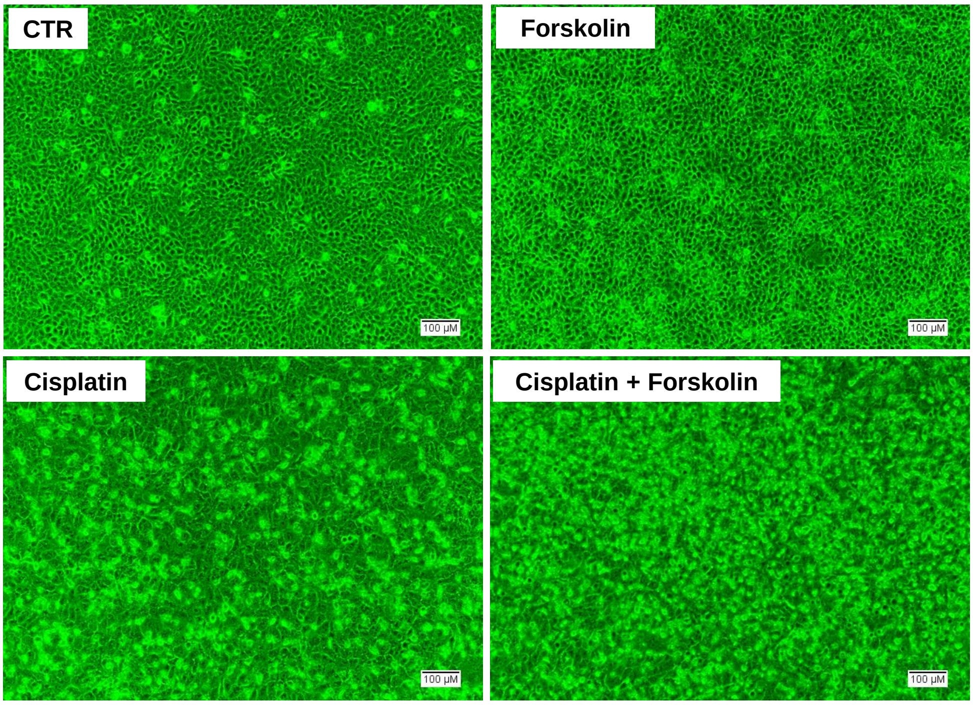

Supplementary Figure 18: PP2A agonist (forskolin) enhanced cisplatin-induced cell proliferation inhibition. Microphotographs of CAL27 cells were treated with forskolin (20 μM) or cisplatin (10 μM) or both for 48 h, scale bar: 100 μm.

Supplemental Figure 19

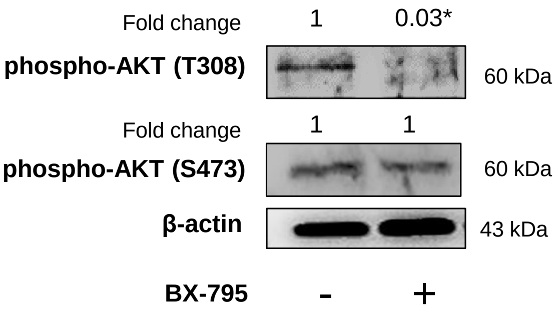

Supplemental Figure 19: PDK1 inhibitor (BX-795) inhibited AKT T308 phosphorylation without affecting AKT S473 phosphorylation. CAL27 cells were treated with BX-795 (10 μM) for 48 h and cell lysates were analyzed by western blot. The target bands were exposed, and densitometry was performed as fold change ratio from the mean of three independent experiments. *t*-test, $*P < 0.05$ vs. the control group.

Supplemental Figure 20

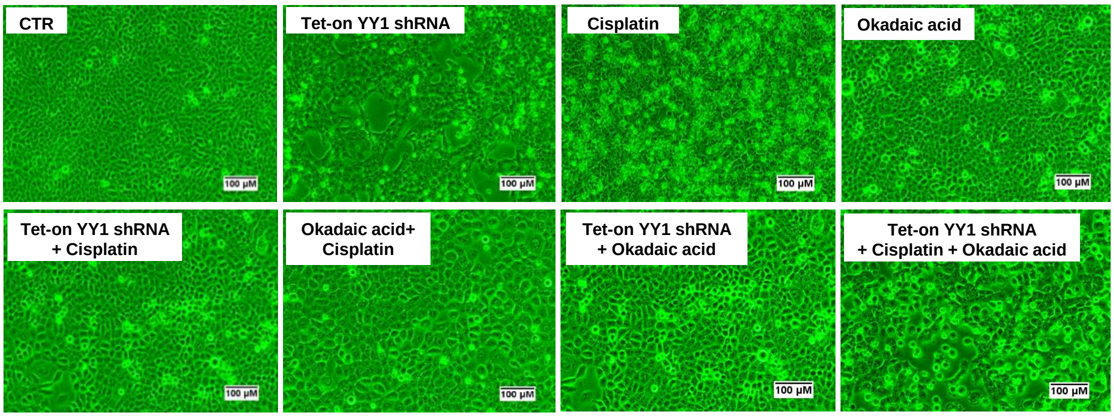

Supplemental Figure 20: PP2A inhibitor (OA) rescued YY1 knockdown or cisplatin or combination of YY1 knockdown and cisplatin-induced cell proliferation inhibition. Microphotographs of CAL27 cells stably transfected with Tet-on YY1 shRNA were exposed to either Doxycycline (100 nM) or okadaic acid (15 nM) or cisplatin (10 μM) or both for 48 h, scale bar: 100 μm.

Supplemental Figure 21

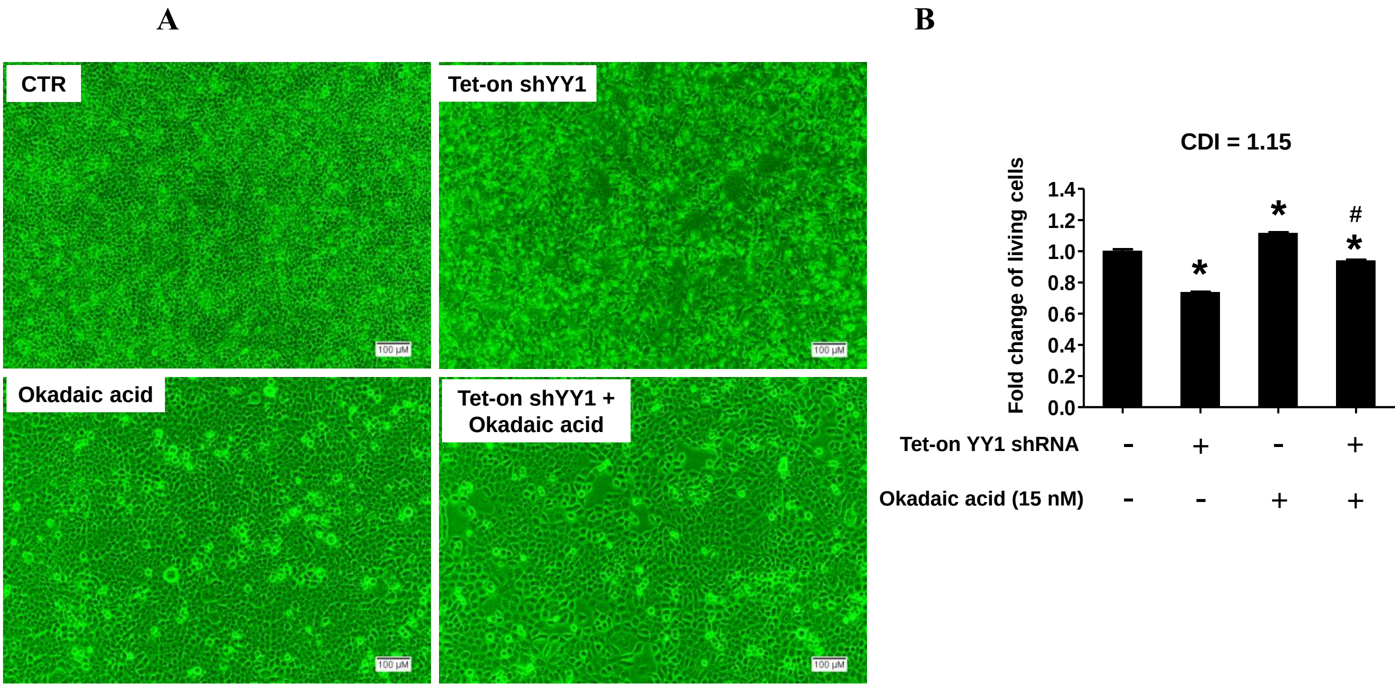

Supplemental Figure 21: PP2A inhibitor (okadaic acid) rescued YY1 knockdown-induced cell proliferation inhibition. CAL27 cells stably transfected with Tet-on YY1 shRNA were exposed to either Doxycycline (100 nM) or okadaic acid (15 nM) or both for 48 h. (A) Microphotographs of CAL27 cells after different treatment, scale bar: 100 µm. (B) CAL27 cells were subjected to CCK-8 assay (n = 4). One-way ANOVA: $^{*}P < 0.05$ vs. the control group; $^{\#}P < 0.05$ vs. YY1 knockdown or okadaic acid group. (Bars: mean ± SD)

Supplemental Figure 22

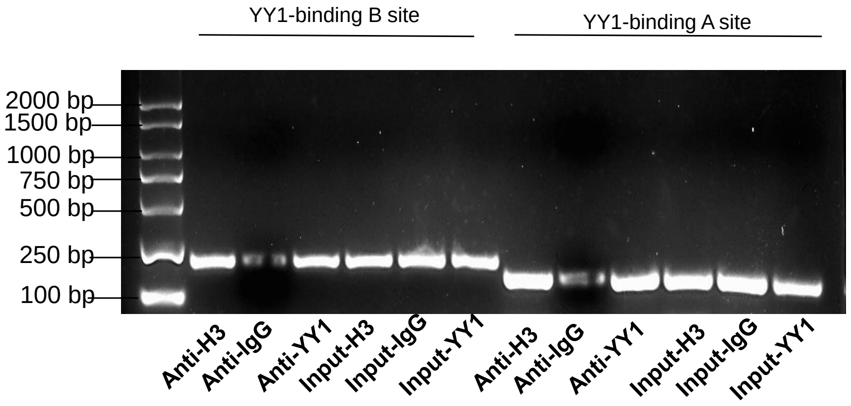

Supplementary Figure 22: YY1 bound to PPP2CA promoter *in vivo*. ChIP assays were performed in CAL27 cells with anti-YY1, anti-H3 or anti-IgG antibodies and with primers amplifying the -1464/-1303 and -1195/-994 region of the PPP2CA promoter containing YY1-binding sites A or B, respectively. ChIP samples were assessed by standard PCR and DNA agarose gel electrophoresis.

Supplemental Figure 23

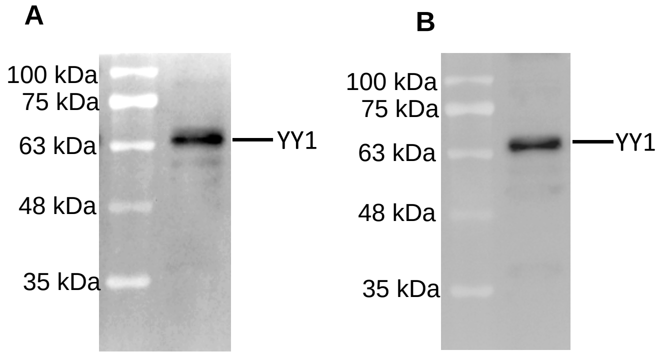

Supplementary Figure 23: Confirmation of YY1 antibodies. CAL27 cell lysates were analyzed by Western blot. A: YY1 antibody from Cell Signaling Technology; B: YY1 antibody from Santa Cruz.

Supplemental Figure 24

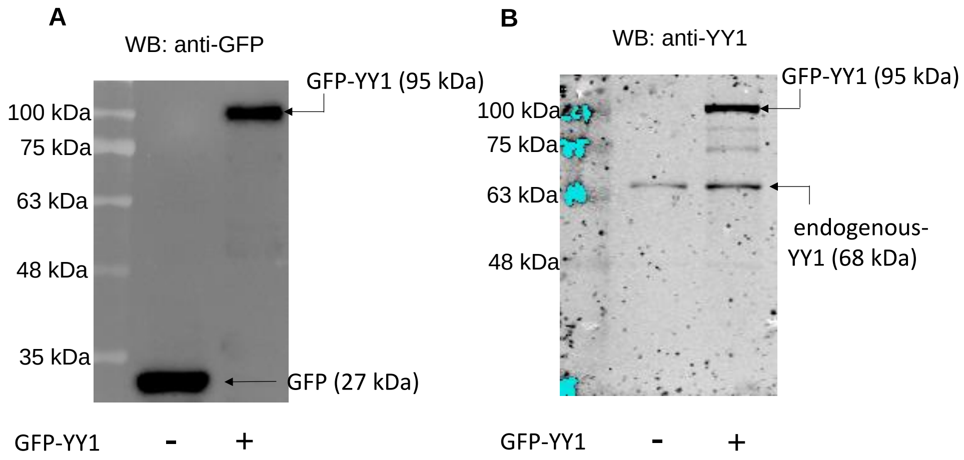

Supplementary Figure 24: Confirmation of YY1 antibodies. CAL27 cells stably transfected with either GFP or GFP-YY1 lentivirus. Cell lysates were analyzed by Western blot. A: GFP antibody from Cell Signaling Technology; B: YY1 antibody from Santa Cruz.
